# Challenging thermodynamics: combining immiscible elements in a single-phase nano-ceramic

**DOI:** 10.1038/s41467-024-45413-w

**Published:** 2024-02-07

**Authors:** Shuo Liu, Chaochao Dun, Qike Jiang, Zhengxi Xuan, Feipeng Yang, Jinghua Guo, Jeffrey J. Urban, Mark T. Swihart

**Affiliations:** 1 Department of Chemical and Biological Engineering, University at Buffalo, The State University of New York, Buffalo, NY 14260 USA; 2 The Molecular Foundry, Lawrence Berkeley National Laboratory, Berkeley, CA 94720 USA; 3 Instrumentation and Service Center for Physical Sciences, Westlake University, Hangzhou, Zhejiang 310024 China; 4 RENEW Institute, University at Buffalo, The State University of New York, Buffalo, NY 14260 USA; 5 Advanced Light Source, Lawrence Berkeley National Laboratory, Berkeley, CA 94720 USA

**Keywords:** Chemical engineering, Nanoparticles, Nanoparticles

## Abstract

The Hume-Rothery rules governing solid-state miscibility limit the compositional space for new inorganic material discovery. Here, we report a non-equilibrium, one-step, and scalable flame synthesis method to overcome thermodynamic limits and incorporate immiscible elements into single phase ceramic nanoshells. Starting from prototype examples including (NiMg)O, (NiAl)O_x_, and (NiZr)O_x_, we then extend this method to a broad range of Ni-containing ceramic solid solutions, and finally to general binary combinations of elements. Furthermore, we report an “encapsulated exsolution” phenomenon observed upon reducing the metastable porous (Ni_0.07_Al_0.93_)O_x_ to create ultra-stable Ni nanoparticles embedded within the walls of porous Al_2_O_3_ nanoshells. This nanoconfined structure demonstrated high sintering resistance during 640 h of catalysis of CO_2_ reforming of methane, maintaining constant 96% CH_4_ and CO_2_ conversion at 800 °C and dramatically outperforming conventional catalysts. Our findings could greatly expand opportunities to develop novel inorganic energy, structural, and functional materials.

## Abstract (web summary)

Elemental immiscibility limits the development of solid solution materials. Here, authors create a nonequilibrium flame aerosol method to mix nearly any pair of metal elements in a single-phase nano-ceramic. Also, an exsolution behavior is presented to produce active and stable nanoparticles.

## Introduction

Heteroatom doping of one element into another crystal lattice can introduce active sites, change electronic structure, modify coordination environment, and alter defect density. Thus, it is a general and widely used approach to optimizing material function for broad application areas such as catalysis^1^, photovoltaics^2^, and electrochemistry^3^. However, incompatibility of elements limits the dissolution of foreign atoms, leaving a narrow range of thermodynamically-stable solid solutions. The thermodynamic constraints are described by the Hume-Rothery rules, which predict the miscibility of solute-solvent solids based upon similarity in crystal structure, atomic radii, valency, and electronegativity. These factors vary widely for different elements (Supplementary Fig. 1), which greatly limits the compositional space for material discovery. For example, (NiMg)O solid solution-derived catalysts show excellent performance for CO_2_ reforming of methane^4^. Unfortunately, immiscibility between NiO and other metal oxides greatly limits the development of other Ni-based catalysts by a similar route.

Conventional wet-chemistry or chemical vapor deposition methods may not provide access to certain phases of materials, making synthesis of kinetically-stable but non-equilibrium forms of matter inaccessible. For example, the miscibility gap often leads to undesirable phase segregation in the commonly adopted co-precipitation method, as solute atoms aggregate during slow nucleation and growth processes. Furthermore, fabrication of nano-ceramic solid solutions is more challenging than metal alloys due to the broader array of crystal lattices adopted by ceramics relative to simple metals. Recently, researchers have developed some non-equilibrium synthesis strategies, such as high-temperature shockwave^5^, laser scanning ablation^6^, and electrical explosion^7^, where the material forms in milliseconds or even nanoseconds. A fast material formation process can prevent atom diffusion and leave immiscible elements mixed in a metastable alloy phase that cannot be achieved using conventional equilibrium synthesis methods^8^. However, most of the non-equilibrium synthesis methods rely on extreme conditions, high energy input, or a specific substrate to load precursor salts and products, features that introduce barriers to scale-up.

Flame aerosol processing, dating back to prehistoric civilizations in which soot was used as a pigment, has been the most common technology for industrial production of nanoparticles such as carbon black, TiO_2_, and fumed silica^9^. Recently, it has been extended to fabrication of advanced nanomaterials^10–13^, mainly focused on modifying particle size, morphology, or crystallinity. The ability to overcome elemental thermodynamic immiscibility in such a scalable method is rare. In this research, we present a general flame aerosol strategy to integrate immiscible elements into a single nano-ceramic phase, based on a modified flame reactor^14^. We also template mesopores in (NiAl)O_x_ solid solution nanoshells through an evaporation-driven micelle self-assembly process^15^ and treat this material under reducing conditions to generate active Ni nanoparticles embedded in porous Al_2_O_3_. This resulting nanoconfined structure exhibits ultrahigh activity and stability as a catalyst for CO_2_ reforming of methane.

## Results

### Non-equilibrium flame synthesis of ceramic solid solution nanoshells

**Fig. 1:**
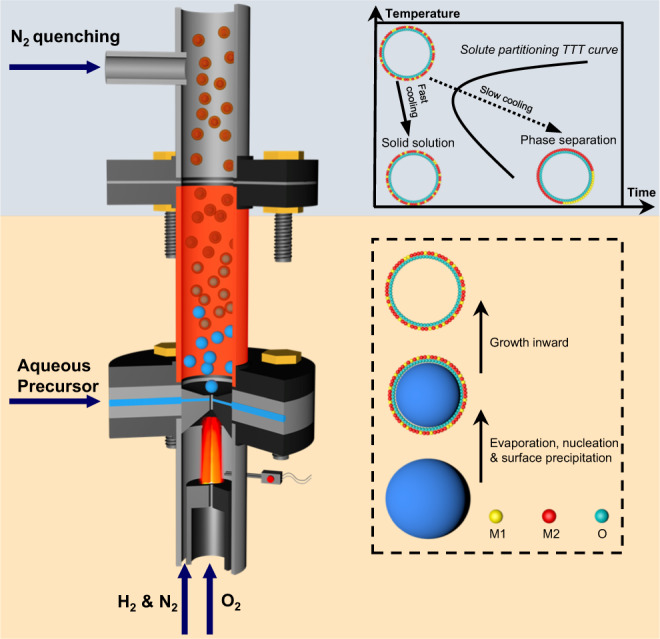
Schematic of the non-equilibrium flame aerosol process producing ceramic solid solution nanoshells via an evaporation driven droplet-to-particle conversion followed by N_2_ quenching. M1 and M2 represent metal elements used in aqueous inorganic salt solutions as precursors.

Detailed flame synthesis procedures are described in the Methods Section (Supplementary Figs. 2, 3). The non-equilibrium synthesis concept presented mainly proceeds by a droplet-to-particle conversion process. As shown in Fig. 1, an aqueous precursor was shear-atomized into droplets of a few micrometers diameter^16,17^, from which solvent evaporates in milliseconds^18,19^. As a result, the immiscible metal atoms are trapped in a metastable phase, forming a uniform ceramic solid solution. The rapid droplet-to-particle conversion tends to generate a hollow nanoshell morphology^20,21^. The solid material formed at the droplet surface grows inwardly as water diffuses out, finally locking in the hollow sphere morphology. Similar to the quenching of alloys, rapid N_2_-quenching prevents phase separation by avoiding the nose point of the solute partitioning TTT curve.

### Comparison between non-equilibrium (flame synthesis) and equilibrium (co-precipitation) synthesis methods

**Fig. 2:**
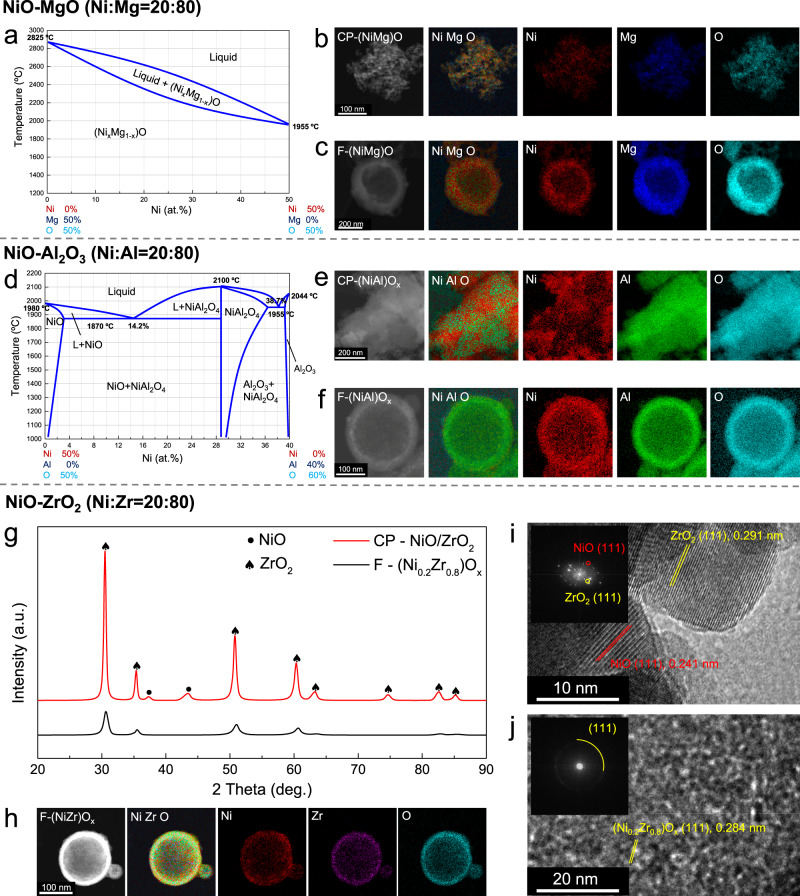
Typical NiO-MgO, NiO-Al_2_O_3_, and NiO-ZrO_2_ materials synthesized by co-precipitation (CP-) and flame aerosol (F-) methods, which are miscible, partly miscible, and immiscible, respectively. **a** NiO-MgO phase diagram^49^; **b**, **c** HAADF-STEM and elemental maps of CP-(NiMg)O_x_ and F-(NiMg)O_x_; **d** NiO-Al_2_O_3_ phase diagram^50^; **e**, **f** HAADF-STEM and elemental maps of CP-(NiAl)O_x_ and F-(NiAl)O_x_; **g** Rietveld refined XRD patterns of CP-NiO/ZrO_2_ and F-(NiZr)O_x_; **h** HAADF-STEM and elemental maps of F-(NiZr)O_x_; **i**, **j** HRTEM images and corresponding FFT patterns of CP-(NiZr)O_x_ and F-(NiZr)O_x_.

We first compare the flame synthesis (F-) with a conventional co-precipitation (CP-) method for three prototypical nickel-containing oxides: NiO-MgO, NiO-Al_2_O_3_, and NiO-ZrO_2_, which are thermodynamically miscible, partially miscible and immiscible, respectively. As expected, for the NiO-MgO system that satisfies the Hume-Rothery rules (Fig. 2a), both F- and CP- based methods produced homogeneous (Ni_0.2_Mg_0.8_)O solid solutions, as confirmed by high-angle annular dark-field scanning transmission electron microscopy (HAADF-STEM) with elemental mapping by energy-dispersive x-ray spectroscopy (EDS), which showed homogeneous elemental distributions of Ni and Mg (Fig. 2b, c). X-ray diffraction (XRD) patterns showed a single rock-salt phase (Supplementary Fig. 5a). For the NiO-Al_2_O_3_ system which can form a NiAl_2_O_4_ spinel phase, the CP-NiO-Al_2_O_3_ material exhibited obvious phase segregation (Fig. 2d, Supplementary Fig. 4), as reflected by a nonuniform elemental distribution (Fig. 2e) and separate NiO, and NiAl_2_O_4_ spinel phases, possibly accompanied by some Al_2_O_3_, in XRD (Supplementary Fig. 5b). In contrast, the flame synthesis method produced a homogeneous F-(Ni_0.2_Al_0.8_)O_x_ solid solution (Fig. 2f) without separated NiO or NiAl_2_O_4_ phases (Supplementary Fig. 5b).

A similar phenomenon was observed for the thermodynamically immiscible NiO-ZrO_2_ system, whose phase diagram has not been published. Conventional co-precipitation failed to dope Ni into the ZrO_2_ lattice, instead yielding separate NiO and ZrO_2_ phases, as shown in the XRD pattern of CP-NiO/ZrO_2_ in which characteristic NiO peaks at 37.2° and 43.5° were detected (Fig. 2g, Supplementary Table 1). In contrast, the F-(Ni_0.2_Zr_0.8_)O_x_ exhibited a single phase without NiO peaks (Fig. 2g, Supplementary Table 2). Compared to the ZrO_2_ peaks in CP-NiO/ZrO_2_, the slight peak shifts of F-(Ni_0.2_Zr_0.8_)O_x_ also demonstrated that Ni was incorporated in the ZrO_2_ lattice, altering the cell parameter. Meanwhile, the F-(Ni_0.2_Zr_0.8_)O_x_ solid solution showed a homogeneous elemental distribution (Fig. 2h), while an inhomogeneous distribution of the elements was evident in CP-NiO/ZrO_2_ (Supplementary Fig. 6). High-resolution transmission electron microscopy with fast-Fourier-transform (FFT) analysis revealed nanoscale phase segregation in CP-NiO/ZrO_2_ (Fig. 2i). Consistent with XRD analysis, different NiO and ZrO_2_ grains were observed. In contrast, the (111) diffraction spots in F-(Ni_0.2_Zr_0.8_)O_x_ formed a circle of fixed diameter (Fig. 2j), indicating nanocrystalline grains of the same phase and lattice constant. These results clearly demonstrate the ability of the flame-based synthesis to overcome immiscibility predicted by violation of the Hume-Rothery rules. Synthesizing single-phase solid solutions in the NiO-ZrO_2_ system poses a significant challenge due to the miscibility gap arising from their dramatic difference in atomic radius (1.62 vs 2.16 Å), preferred valence (+2 vs +4), electronegativity (1.91 vs 1.33), and crystal structure (rock-salt vs tetragonal). However, achieving this goal would powerfully demonstrate that the flame synthesis method can produce metastable materials regardless of elemental miscibility. Thus, we intentionally selected this prototype system to establish the concept before extending it to other Ni-based and general combinations.

In addition, materials prepared by co-precipitation exhibited a relatively high degree of crystallinity and large grains, while the flame synthesized solid solutions showed a hollow nanoshell morphology with a polycrystalline structure of nanoscale grains. These thin shells consisted of numerous grains and abundant grain boundaries, which can play critical roles in increasing performance in catalysis and related applications^22,23^.

### Extension to numerous Ni-containing ceramic solid solution nanoshells

**Fig. 3:**
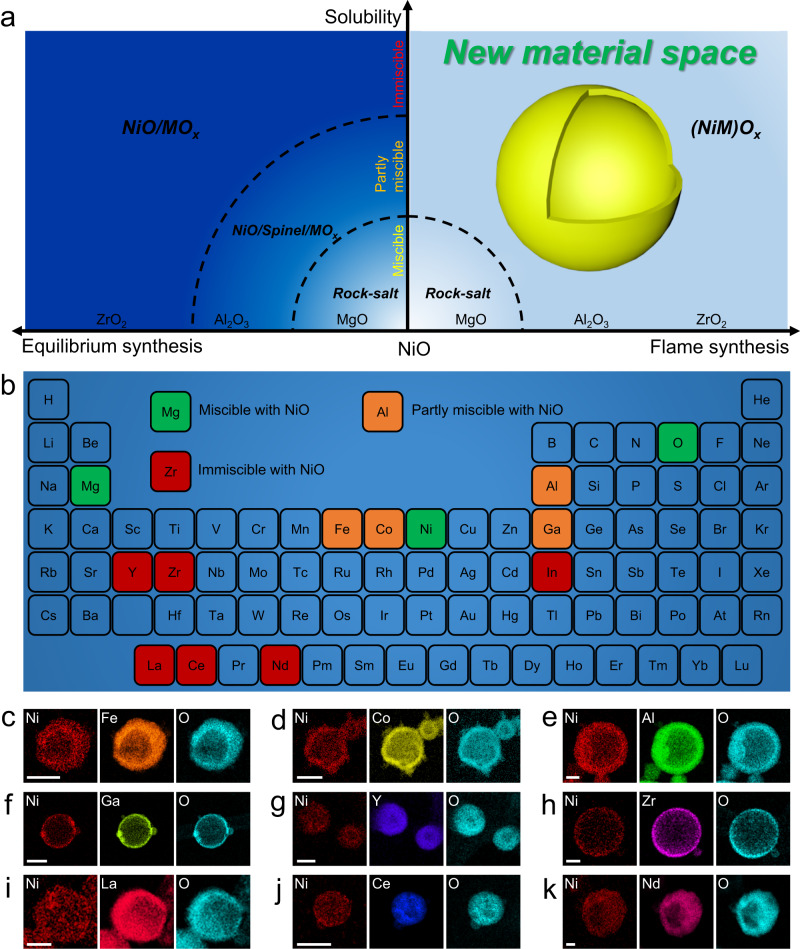
Palette of Ni-containing ceramic solid solution nanoshells. **a** Illustration of the expansion of material space, in which partly miscible and immiscible pairs exhibit phase separation in equilibrium synthesis but form a single phase using the current flame synthesis method; **b** Miscibility of NiO with other metal oxides; Elemental maps of the flame synthesized. **c** (Ni_0.1_Fe_0.9_)O_x_; **d** (Ni_0.1_Co_0.9_)O_x_; **e** (Ni_0.1_Al_0.9_)O_x_; **f** (Ni_0.1_Ga_0.9_)O_x_; **g** (Ni_0.1_Y_0.9_)O_x_; **h** (Ni_0.1_Zr_0.9_)O_x_; **i** (Ni_0.1_La_0.9_)O_x_; **j** (Ni_0.1_Ce_0.9_)O_x_ and **k** (Ni_0.1_Nd_0.9_)O_x_, scale bars are 100 nm. Corresponding optical images, TEM images, XRD patterns, EDS spectra, and XPS profiles are shown in Supplementary Figs. 8–15.

Overcoming elemental immiscibility would greatly expand the accessible material space relative to conventional approaches (Fig. 3a). Thus, a diverse palette of Ni-containing ceramic solid solution nanoshells was created to demonstrate the generality of the non-equilibrium flame synthesis method, whose solvent metal oxides are partly miscible or immiscible with NiO (Fig. 3b). The maps exhibited homogeneous elemental distributions without any Ni aggregation (Fig. 3c–k), and the XRD patterns of each of the Ni-containing solid solutions matched that of the solvent metal oxide, without phase separation (Supplementary Fig. 11), indicating that all materials formed homogeneous ceramic solid solution nanoshells.

For the (Ni_0.1_In_0.9_)O_x_, smaller nanoparticles that are characteristic of formation by a gas-to-particle route were observed around the nanoshell (Supplementary Figs. 7, 9j)^11^, but the Ni was still well incorporated into the In_2_O_3_ lattice in these nanoparticles, demonstrating that both droplet-to-particle and gas-to-particle routes in the current flame aerosol process can generate homogeneous ceramic solid solutions. Detailed information related to material appearance, crystal structure, composition, and elemental state is provided in Supplementary Figs. 8–15.

### General ceramic solid solution nanoshells beyond NiO

**Fig. 4:**
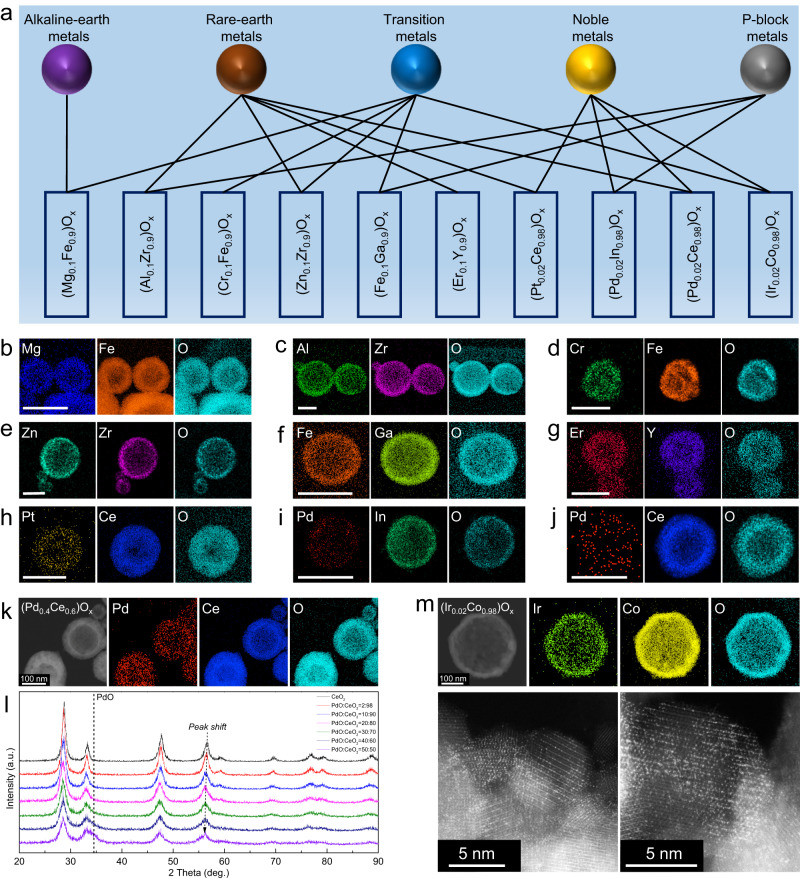
Library of general ceramic solid solution nanoshells. **a** Illustration of the generality of current flame synthesis method by combining elements from different regions of the periodic table. Elemental maps of flame synthesized (**b**) (Mg_0.1_Fe_0.9_)O_x_; **c** (Al_0.1_Zr_0.9_)O_x_; **d** (Cr_0.1_Fe_0.9_)O_x_; **e** (Zn_0.1_Zr_0.9_)O_x_; **f** (Fe_0.1_Ga_0.9_)O_x_; **g** (Er_0.1_Y_0.9_)O_x_; **h** (Pt_0.02_Ce_0.98_)O_x_; **i** (Pd_0.02_In_0.98_)O_x_ and **j** (Pd_0.02_Ce_0.98_)O_x_, scale bar 100 nm; **k** HAAFD-elemental maps of the (Pd_0.4_Ce_0.6_)O_x_; **l** XRD diffraction patterns of (PdCe)O_x_ solid solutions with increasing Pd content from 0 to 50 mol.%; **m** HAADF-STEM elemental maps and AC-STEM of (Ir_0.02_Co_0.98_)O_x_. Corresponding optical images, TEM images, XRD diffraction patterns, EDS spectra, and XPS profiles are shown in Supplementary Figs. 16–21.

The successful mixing NiO into a broad range of metal oxides motivated us to further investigate the generality of this approach. As shown in Fig. 4a, to demonstrate the generality as much as possible, we selected pairs of elements from different regions of the periodic table, excluding radioactive elements. The representative library of ceramic solid solution materials comprised (MgFe)O_x_, (AlZr)O_x_, (CrFe)O_x_, (ZnZr)O_x_, (FeGa)O_x_, (ErY)O_x_, (PtCe)O_x_, (PdIn)O_x_, (PdCe)O_x_, and (IrCo)O_x_, which are of interest for applications including heavy metal ion adsorption^24^, dental filling materials^25^, photoconductors^26^, catalysis of CO_2_ hydrogenation to methanol^27^, dielectric material^28^, up-conversion emission^29^, catalysis of CO oxidation^30^, H_2_ sensors^31^, catalytic methane combustion^32^, and oxygen evolution reaction under acidic conditions^33^, respectively. All of them formed homogeneous ceramic solid solution nanoshell structures. STEM elemental mapping confirmed the homogeneity of all the solid solutions (Fig. 4b–j, m). XRD patterns further confirmed that the solutes were incorporated into the solvents’ crystal lattices, with most particles adopting a hollow nanoshell morphology in these systems (Supplementary Figs. 17, 18). Detailed information related to material appearance, composition, and elemental state is provided in Supplementary Figs. 16 and  19–21.

We further investigated the extent to which the ratio between solute and solvent elements could be tuned. Similar to the (NiCe)O_x_ solid solution, PdO-CeO_2_ was chosen as a model system due to its immiscibility and broad applications in catalysis^34^. (PdCe)O_x_ solid solution materials were synthesized with increasing Pd content from 0 to 50 mol.% in the precursor. The XRD results show only a single fluorite phase (Fig. 4l) with minimal evidence of a PdO peak, consistent with the uniform distribution of Pd, Ce, O in the (Pd_0.4_Ce_0.6_)O_x_ solid solution nanoshell (Fig. 4k). The slight peak shift in XRD peak position reflected incorporation of more Pd upon increasing Pd content in the precursor, accompanied by a decrease in the crystallinity after doping (Fig. 4l, Supplementary Fig. 11, Supplementary Fig. 15 and Supplementary Fig. 18). These results suggested that the atomic ratio in these systems can be readily tuned, and high solubility is likely to be achieved in ceramic solid solutions by our non-equilibrium flame synthesis. Moreover, although it is not the main focus of this work, we note that doping highly dispersed noble metal cation active sites (like Pd, Pt, Ir) into the support lattice is also feasible. To confirm it, aberration corrected scanning transmission electron microscopy (AC-STEM) revealed the crystal lattice and elemental distribution of (Ir_0.02_Co_0.98_)O_x_, demonstrating a high concentration of Ir active sites at the lattice positions of Co and atomically dispersed in the Co_3_O_4_ support (Fig. 4m).

### “Encapsulated exsolution” phenomenon

**Fig. 5:**
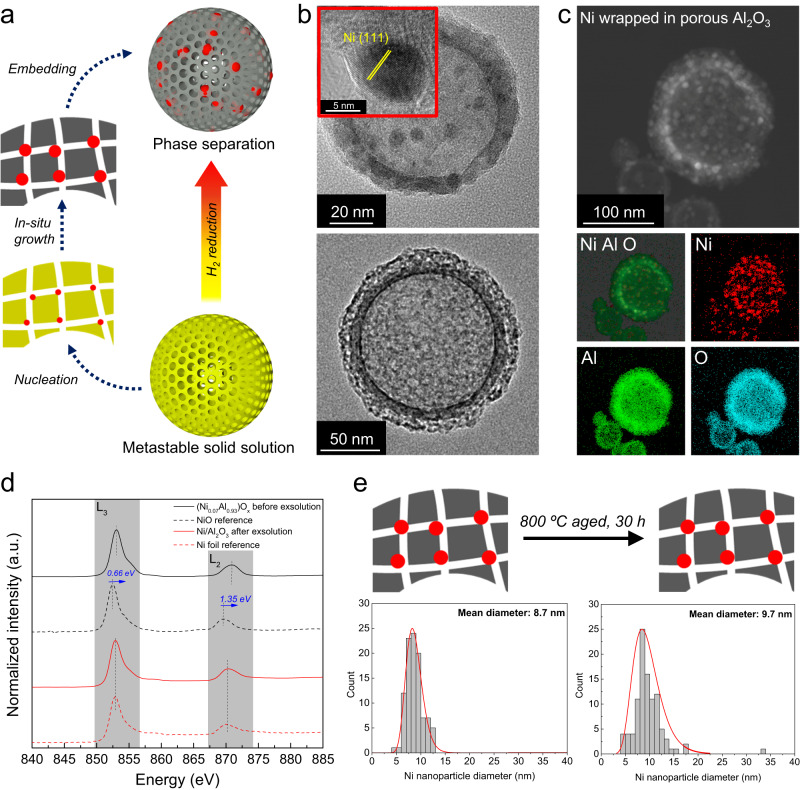
Encapsulated exsolution behavior. **a** Schematic of Ni nanoparticles exsolved from hollow and porous (Ni_0.07_Al_0.93_)O_x_ solid solution in H_2_ (red-Ni, gray-Al_2_O_3_); **b** TEM images of as-synthesized (Ni_0.07_Al_0.93_)O_x_ and exsolved F-Ni/Al_2_O_3_; **c** HAADF-STEM and elemental maps of the exsolved Ni/Al_2_O_3_; **d** Ni L-edge XANES spectra; **e** Ni size distribution curves of Ni/Al_2_O_3_ before and after aging at 800 °C for 30 h, obtained from statistics of 100 Ni nanoparticles in TEM images (red-Ni, gray-Al_2_O_3_).

The (Ni_0.07_Al_0.93_)O_x_ was selected as a model material for further exploration. We found that the oxygen vacancy concentration of F-(Ni_0.07_Al_0.93_)O_x_ was twice that of CP-(Ni_0.07_Al_0.93_)O_x_ (Supplementary Fig. 22)^35^. For catalysis applications, we adopted an evaporation-driven micelle self-assembly method to template mesopores in the (Ni_0.07_Al_0.93_)O_x_ in-situ during synthesis, creating mesoporous (Ni_0.07_Al_0.93_)O_x_ nanoshells (Supplementary Figs. 23, 24)^15^. Using this mesoporous metastable binary oxide, we discovered an “encapsulated exsolution” phenomenon, as shown in Fig. 5. Typical exsolution behavior involves physical processes of diffusion, reduction, nucleation and growth, and generates active particles on a metal oxide surface as a reducible metal is exsolved from the oxide^36^. However, upon heating the porous (Ni_0.07_Al_0.93_)O_x_ solid solution in H_2_ to reduce Ni, the pores provided internal nucleation sites within the alumina shell. Thus, the Ni diffused towards the pores in the shell rather than exclusively toward the outer surface, finally forming a structure of Ni nanoparticles encapsulated in porous and hollow Al_2_O_3_ (Fig. 5a). Transmission electron microscopy (TEM) images clearly showed that the Ni nanoparticles formed in the hollow Al_2_O_3_ after exsolution, and not primarily on the external surface (Fig. 5b). HAADF-STEM elemental mapping of the exsolved Ni/Al_2_O_3_ further demonstrated the encapsulated structure with a high concentration of Ni nanoparticles well dispersed in the hollow Al_2_O_3_ shell (Fig. 5c). We also measured soft X-ray absorption spectra (XAS) at the Ni L_2,3_-edge in (Ni_0.07_Al_0.93_)O_x_ solid solution and reduced F-Ni/Al_2_O_3_ (Fig. 5d). The significant peak shift of (Ni_0.07_Al_0.93_)O_x_ compared to NiO reference indicates a substantial quantity of Ni^3+^ in (Ni_0.07_Al_0.93_)O_x_, which reflected the high defect density and was consistent with the oxygen vacancy analysis from XPS (Supplementary Fig. 22)^37^. After reduction, the XAS profile of reduced Ni/Al_2_O_3_ overlapped with the Ni foil reference, indicating all the Ni cations were reduced to a metallic state, in agreement with Ni *2p* XPS analysis (Supplementary Fig. 25). XRD patterns also showed the separate Ni metal phase formed after reduction, with weak peaks of γ-Al_2_O_3_ phase also appearing (Supplementary Fig. 26) while the as synthesized (Ni_0.07_Al_0.93_)O_x_ solid solution was amorphous.

This newly demonstrated “encapsulated exsolution” has several obvious advantages over the conventional exsolution process^36^: I. Fast exsolution speed. Exsolution of Ni nanoparticles from the current (NiAl)O_x_ solid solution took less than 4 h at 800 °C in H_2_ reducing atmosphere, while for exsolution of Ni from spinel, in a prior report, most Ni^2+^ remained in the spinel lattice after 7 h at 800 °C^38^. The intrinsic metastability, lattice mismatch between dopant and parent ions, and abundant oxygen vacancies lower the energy barriers to cation diffusion and phase segregation, accelerating the exsolution process^39^. II. Ultrasmall nanoparticle size. The “encapsulated exsolution” yielded small Ni nanoparticles of 8.7 nm (Fig. 5e), dramatically smaller than the Ni nanoparticles generated by conventional exsolution from various spinel and perovskite materials (>30 nm)^40–43^. III. Ultrahigh thermal stability. The nanoconfined structure after exsolution provided powerful sintering resistance. The current exsolved Ni/Al_2_O_3_ was aged at 800 °C for 30 h with only a slight increase in Ni particle size, from 8.7 to 9.7 nm based on TEM analysis (from 9.7 to 13.7 nm by XRD analysis) (Fig. 5e, Supplementary Fig. 26). This is comparable to a recent report of Pt nanoparticles encapsulated in porous Al_2_O_3_^44^. TEM images and elemental mapping demonstrated that the morphology, Ni nanoparticle dispersion, and encapsulated structure of F-Ni/Al_2_O_3_ remained the same after aging (Supplementary Fig. 27). When we increased the calcination temperature to 1000 °C, the Ni average nanoparticle size grew to 22 nm (Supplementary Fig. 28), with the majority of Ni remaining as un-sintered small Ni nanoparticles. At 1200 °C, the hollow structure was still maintained even after the Ni nanoparticles dissolved in α-Al_2_O_3_. This demonstrated that the current “encapsulated exsolution” process can be reversed, and the material could be self-regenerative through a “exsolution⇆dissolution” cycle^45^.

Current exsolution research relies on existing materials. The non-equilibrium flame aerosol process can combine nearly any pair of elements. Thus, any reducible elements (e.g., Co, Ni, Cu, Pt, Pd, Ir, Au, …) can be doped into an oxide host lattice of an irreducible element (e.g., Al, Ce, La, Mg, Zr, Y, In, …), to serve as the exsolution precursors, which dramatically expands the variety of exsolution parent materials beyond perovskites and spinels.

### A prototypical catalysis application in CO_2_ reforming of methane

**Fig. 6:**
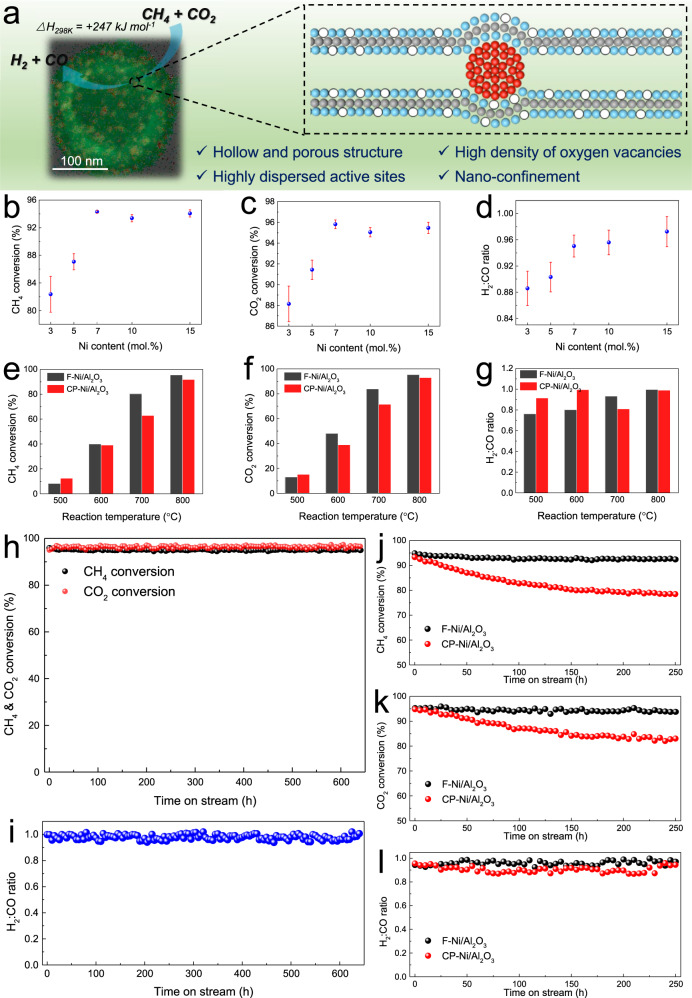
Application of Ni/Al_2_O_3_ in catalysis. **a** Catalyst properties and application in CO_2_ reforming of methane; Activity analysis of the F-Ni/Al_2_O_3_ catalysts with varied Ni content. **b** CH_4_ conversion. **c** CO_2_ conversion, and (**d**) H_2_:CO ratio at 800 °C over 30 h (Corresponding time on stream curves are shown in Supplementary Figs. 29–31; error bars represent one standard deviation of the distribution of 180 values measured over 30 h.); Activity analysis of F-Ni/Al_2_O_3_ and CP-Ni/Al_2_O_3_ catalysts at varied reaction temperatures. **e** CH_4_ conversion. **f** CO_2_ conversion. **g** H_2_:CO ratio; Stability analysis of F-Ni/Al_2_O_3_ catalyst at 800 °C for 640 h. **h** CH_4_ conversion and CO_2_ conversion. **i** H_2_:CO ratio; Stability analysis of F-Ni/Al_2_O_3_ and CP-Ni/Al_2_O_3_ catalysts at 800 °C for 250 h. **j** CH_4_ conversion. **k** CO_2_ conversion. **l** H_2_:CO ratio. Reaction conditions in (**b**–**g** and **j**–**l**): 60 mL min^−1^ total feed gas flow rate, CH_4_:CO_2_:Ar = 1:1:1, 20 mg catalyst loading, 180,000 mL g_cat_^−1^ h^−1^ GHSV, atmospheric pressure. Reaction conditions in (**h**, **l**): 30 mL min^−1^ total feed gas flow rate, CH_4_:CO_2_:Ar = 1:1:1, 30 mg catalyst loading, 60,000 mL g_cat_^−1^ h^−1^ GHSV, atmospheric pressure.

The flame synthesized and exsolved F-Ni/Al_2_O_3_ showed features that are desired in catalysis applications, including their hollow and porous structure that facilitates rapid mass transfer of reactant gases; highly dispersed active sites and abundant oxygen vacancies that increase reactant adsorption and conversion; and the nano-confined structure that impart high sintering resistance to reduce catalyst deactivation. Here, we selected CO_2_ (dry) reforming of methane (CH_4_ + CO_2_ ⇆ 2H_2_ + 2CO), which converts the two most prominent greenhouse gases to a valuable chemical feedstock^46^, as a representative application to examine catalytic performance (Fig. 6a).

The influence of Ni content (3, 5, 7, 10, and 15 mol.%) on catalyst performance was investigated first under continuous time-on-stream tests at 800 °C for 30 h, in which the material with 7 mol.% Ni showed the best activity and stability. Further increasing the Ni content did not improve performance (Fig. 6b–d, Supplementary Figs. 29–31). Then, the activity of the optimized F-Ni/Al_2_O_3_ catalyst was investigated at different reaction temperatures, compared to a conventional CP-Ni/Al_2_O_3_ catalyst prepared by a co-precipitation method with the same 7 mol.% Ni content (Fig. 6e–g). As expected for this endothermic reaction, CH_4_ and CO_2_ conversions increased with increasing reaction temperature. At low temperatures, CH_4_ conversion was lower than CO_2_ conversion and the H_2_/CO ratio was lower than 1 due to occurrence of the reverse water gas shift reaction (CO_2_ + H_2_ ⇆ CO + H_2_O). At 800 °C, F-Ni/Al_2_O_3_ exhibited ~96% CH_4_ and CO_2_ conversions with a H_2_/CO ratio close to 1. In general, F-Ni/Al_2_O_3_ demonstrated greater catalytic activity than CP-Ni/Al_2_O_3_.

To date, CO_2_ reforming of methane has not reached large-scale commercial application, and the major barrier has been catalyst deactivation at high reaction temperature^47^. Thus, we investigated the stability of F-Ni/Al_2_O_3_ at 800 °C with gas hourly space velocity (GHSV) of 60,000 mL g_cat_^-1^ h^-1^. The F-Ni/Al_2_O_3_ catalyst maintained constant ~96% CH_4_ and CO_2_ conversions for 640 h (27 days), with a H_2_/CO ratio close to 1 (Fig. 6h, i). Furthermore, we increased the GHSV by a factor of 3 to 180,000 mL g_cat_^-1^ h^-1^, and the F-Ni/Al_2_O_3_ catalyst still maintained the same activity and stability (Fig. 6j–l). In contrast, under the same reaction conditions, the conventional CP-Ni/Al_2_O_3_ catalyst exhibited decreasing CH_4_ and CO_2_ conversions, suggesting catalyst deactivation. Typically, most approaches for designing stable catalysts lead to activity-stability trade-offs, in which high stability is achieved at the expense of activity^44^. Encouragingly, the F-Ni/Al_2_O_3_ catalyst exhibited both long-term stability and ultra-high activity, demonstrating advantages of the non-equilibrium flame synthesis method compared with previously reported methods of producing Ni/Al_2_O_3_ catalysts^48^.

## Discussion

This research establishes a general methodology to circumvent thermodynamic immiscibility to create a broad array of homogeneous metastable solid solution nano-ceramics. These can enable new properties and applications either in their as-synthesized metastable state, or by serving as precursors for nano-phase separated systems. This class of materials exhibits exploitable features including highly dispersed solute atoms, hollow nanoshell morphology, and high defect density, with controllable characteristics like flexible composition and solute/solvent ratio, templated pore structure, and tailored phase-separation during post-processing, enabling rational design of material properties for targeted applications, such as CO_2_ reforming of methane. The intrinsic scalability, single-step and continuous operation, and relatively low cost of the aerosol processing approach employed here strongly suggest that it can be economically applied for large-scale production in industry, paving the way for widespread development and application of inorganic nanomaterials in fields including sensing, energy storage, and catalysis.

## Methods

### Chemicals

Precursor salts of Ni(NO_3_)_2_•6H_2_O (99%), Mg(NO_3_)_2_•6H_2_O (99 + %), Al(NO_3_)_3_•9H_2_O (99 + %), ZrO(NO_3_)_2_•H_2_O (99.5%), Co(NO_3_)_2_•6H_2_O (99 + %), Ga(NO_3_)_3_•H_2_O (99.99%), Ce(NO_3_)_3_•6H_2_O (99.5%), and Cr(NO_3_)_3_•9H_2_O (99%) were purchased from Acros Organics; In(NO_3_)_3_•H_2_O (99.99%), Nd(NO_3_)_3_•6H_2_O (99.9%), Er(NO_3_)_3_•5H_2_O (99.9%), and Pt(NO_3_)_4_ solution (Pt 15 w/w) were purchased from Alfa Asear; Fe(NO_3_)_3_•9H_2_O was purchased from Fisher Chemical; Zn(NO_3_)_2_•6H_2_O (98%) was purchased from Sigma Aldrich; Pd(NO_3_)_2_•H_2_O (99.8%, Pd 39% min) and IrCl_3_•3H_2_O (Ir 53–56%) were purchased from Thermo Scientific. NaOH pellets were purchased from Sigma Aldrich. Hexadecyltrimethylammonium bromide (CTAB, 99 + %) and HCl (37%) were purchased from Acros Organics. Ethanol (200 proof) was obtained from Decon Labs, Inc.

### Flame aerosol synthesis

A flowing gas mixture of 8.5 L min^-1^ H_2_, 15 L min^-1^ N_2_, and 7.5 L min^-1^ O_2_ was ignited to form an inverted diffusion flame. The hot combustion products passed through a converging-diverging nozzle (drill Ø0.1111 inch, #34) to form a sonic velocity hot stream. An aqueous precursor solution of desired composition was prepared by dissolving metal salts in water at a total concentration of 20 mM and desired molar ratio. When the temperature and pressure in the reactor stabilized, the liquid precursor was injected into the throat section of the nozzle (drill Ø0.1040 inch, #37) by a peristaltic pump at 300 mL h^-1^ flow rate, where it was atomized by the high-velocity gas stream. These operating conditions produce a typical temperature of ~800 °C in the reactor, downstream of the nozzle. In this high temperature reaction zone, each droplet evaporated and a ceramic solid solution nanoshell formed from each droplet. Downstream of the reaction zone, the product was immediately cooled by a 140 L min^-1^ N_2_ flow to prevent phase separation and particle sintering. The product was collected on a filter membrane (Millipore Durapore PVDF, 0.22 μm nominal pore size, 29.3 cm diameter). A downstream vacuum pump provided control of pressure in the reactor system. The current laboratory-scale reactor yields roughly 1 g per hour for the materials reported here.

### Co-precipitation synthesis

The precursor solution was prepared by dissolving two inorganic salts in water with total concentration of 100 mM and desired molar ratio. Then, 1 M NaOH solution was added dropwise into the precursor solution under vigorous stirring until the pH value reached 11. The solution was stirred at 80 °C for 2 h. After that, the intermediate product was collected by vacuum filtration, washing, and drying at 100 °C overnight. Finally, the sample was calcined at 550 °C in air for 4 h. The CP-Ni/Al_2_O_3_ catalyst was synthesized by the same procedures with a Ni to Al molar ratio of 0.07:0.93 in the precursor. The catalyst was reduced by 20 mL min^−1^ H_2_ at 800 °C for 4 h before use as a catalyst.

### Synthesis of porous (Ni_0.07_Al_0.93_)O_x_ solid solution

The precursor solution was prepared by dissolving Ni(NO_3_)_2_, Al(NO_3_)_3_, and CTAB into a mixed solvent of water and ethanol. The Al(NO_3_)_3_ concentration was 30 mM with Ni to Al molar ratio of 0.07:0.93 and CTAB to Al molar ratio of 0.25:1. HCl was added to the precursor solution to reach a pH value of 2. The H_2_ flow rate was changed to 6.5 L min^-1^, which generated a temperature of ~700 °C in the reactor chamber. Other procedures were as described above for the general flame synthesis. The intermediate product collected after flame synthesis was calcined at 550 °C in air for 4 h to remove the micelle template.

### Ni encapsulated exsolution

The porous (Ni_0.07_Al0_.93_)O_x_ solid solution was heated in 20 mL min^−1^ H_2_ reducing atmosphere at 800 °C for 4 h. The material after exsolution was also the F-Ni/Al_2_O_3_ catalyst.

### Material characterizations

High-angle annular dark-field scanning transmission electron microscopy (HAADF-STEM) imaging with elemental mapping by EDS, and live FFT patterns were obtained using a JEOL 2100-F 200 kV field-emission analytical TEM; Aberration corrected scanning transmission electron microscopy (AC-STEM) analysis was performed on a JEOL JEM ARM200F thermal-field emission microscope with a probe spherical aberration (Cs) corrector working at 200 kV; The material morphology was also characterized using a JEOL JEM 2010 TEM; XRD patterns were measured using an X-ray diffractometer (Rigaku Ultima IV) with Cu Kα source (λ = 0.15418 nm). The diffractometer was operated at 40 mA and 40 KV in the 2θ range of 5–90° at a scan speed of 2°/min; XAS were measured at Beamline 7.3.1 at the Advanced Light Source (ALS), Lawrence Berkeley National Laboratory. This is a bending-magnet beamline with a photon energy range from 250 to 1650 eV. The base pressure of the main chamber is below 1 × 10^-9 ^Torr. The TEY signal was obtained by monitoring the sample drain current. All the powder samples were mounted on the sample holder using a carbon tape. To further increase the TEY intensity, silver paste was used to connect the surface of these samples to the metallic sample holder to reduce charging; X-ray photoelectron spectroscopy (XPS, Thermo Fisher, USA) measurements were conducted to analyze the surface metal state and oxygen vacancies. The photoelectron spectrometer system was configured with an Al Kα excitation source with spot size of 400 μm. Before collecting an XPS spectrum, an ion flood source was applied for charge neutralization; N_2_ physisorption measurements (Micromeritics Tri-Star II) were used to characterize pore structure at 77 K. The samples were degassed at 250 °C for 4 h prior to analysis to remove moisture.

### Catalyst tests

Catalyst performance was measured using a continuous fixed-bed flow reactor with an internal diameter of 4 mm at atmospheric pressure. Each catalyst was loaded between quartz wool plugs in the reactor. The catalyst was heated to 800 °C under 20 mL min^-1^ H_2_ flow for 1 h and was kept at 800 °C for 4 h. Then, the reactor was switched to the targeted reaction temperature. The reactant gases (CH_4_:CO_2_:Ar = 1:1:1) at the desired total flow rate were introduced into the reactor to conduct the dry reforming of methane reaction. The effluent gases were analyzed online by a gas chromatograph (SRI 8610) fitted with a packed column (Restek Natural Gas ShinCarbon ST) and a thermal conductivity detector (TCD). The CH_4_ conversion, CO_2_ conversion, and H_2_/CO ratio were calculated based on the following Eqs. (1)–(3):

1 $${X}_{{{CH}}_{4}}=\frac{{F}_{{{in},{CH}}_{4}}-{F}_{{{out},{CH}}_{4}}}{{F}_{{{in},{CH}}_{4}}}\times 100\%$$

2 $${X}_{{{CO}}_{2}}=\frac{{F}_{{{in},{CO}}_{2}}-{F}_{{{out},{CO}}_{2}}}{{F}_{{{in},{CO}}_{2}}}\times 100\%$$

3 $${H}_{2}/{CO}=\frac{{F}_{{{out},H}_{2}}}{{F}_{{out},{CO}}}$$

### Supplementary information

Supplementary Information

Peer Review File

## Data Availability

The data that supports the findings of the study are included in the main text and supplementary information files. Raw data can be obtained from the corresponding author upon request.
