# Supplementary material for: Challenging thermodynamics: combining immiscible elements in a single-phase nano-ceramic
Source: Nat Commun. 2024 Feb 7;15:1167. doi: 10.1038/s41467-024-45413-w (PMC10850329; doi:10.1038/s41467-024-45413-w)
Supplement: Supplementary file 1 — Supplementary Information [file 41467_2024_45413_MOESM1_ESM.pdf]

Supplementary Information for:

Challenging Thermodynamics: Combining Immiscible Elements in a Single-phase Nano-ceramic

Shuo Liu [1], Chaochao Dun [2*], Qike Jiang [3], Zhengxi Xuan [1,4], Feipeng Yang [5], Jinghua Guo [4], Jeffrey J. Urban [2*], and Mark T. Swihart [1,4*]

[1] Department of Chemical and Biological Engineering, University at Buffalo, The State University of New York, Buffalo, NY 14260, USA

[2] The Molecular Foundry, Lawrence Berkeley National Laboratory, Berkeley, CA 94720, USA

[3] Instrumentation and Service Center for Physical Sciences, Westlake University, Hangzhou, Zhejiang, 310024, China

[4] RENEW Institute, University at Buffalo, The State University of New York, Buffalo, NY 14260, USA

[5] Advanced Light Source, Lawrence Berkeley National Laboratory, Berkeley, CA, 94720, USA

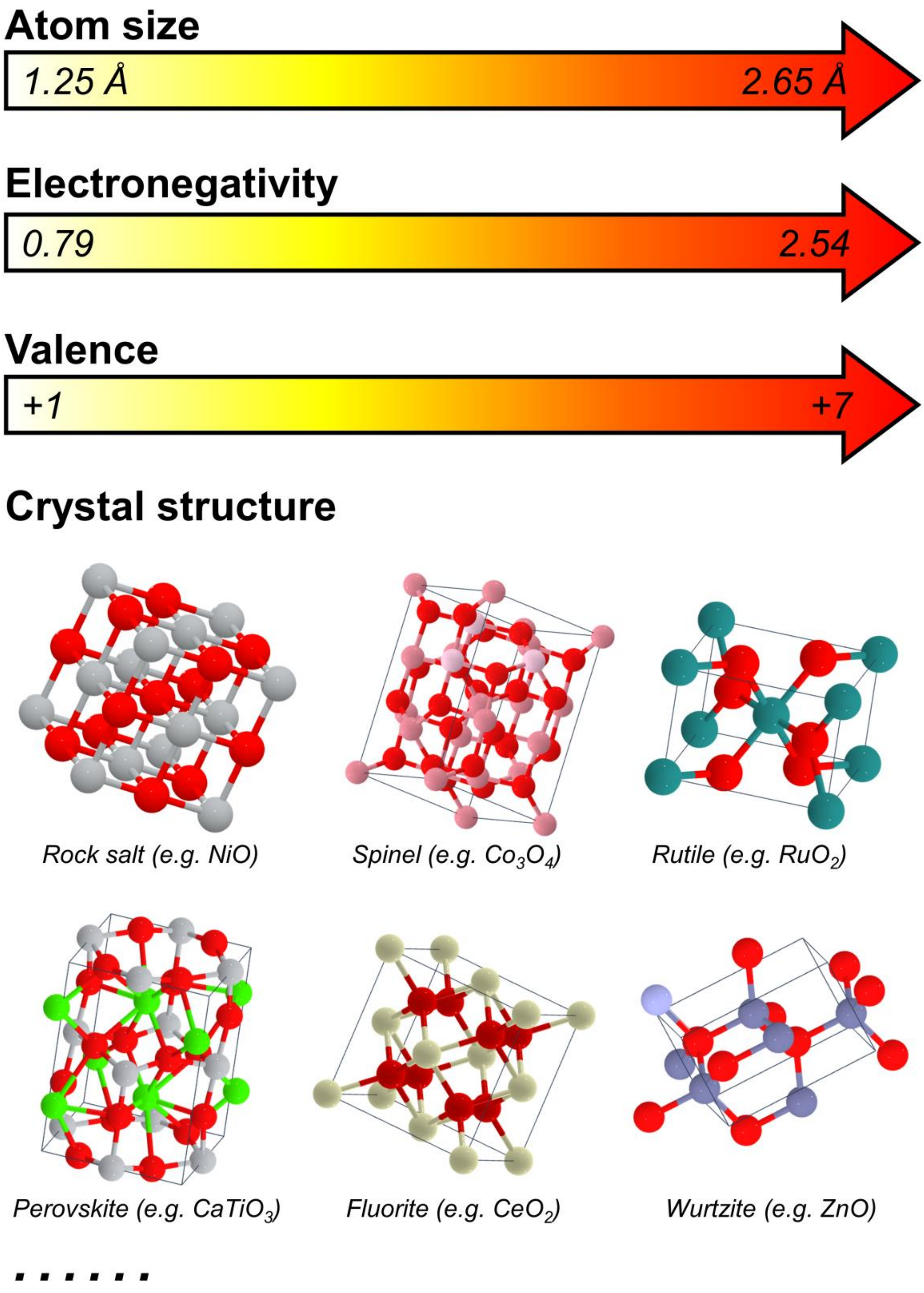

Supplementary Fig. 1. Schematic illustration of the wide range of values of characteristics of elements that govern their miscibility as cations in oxide phases.

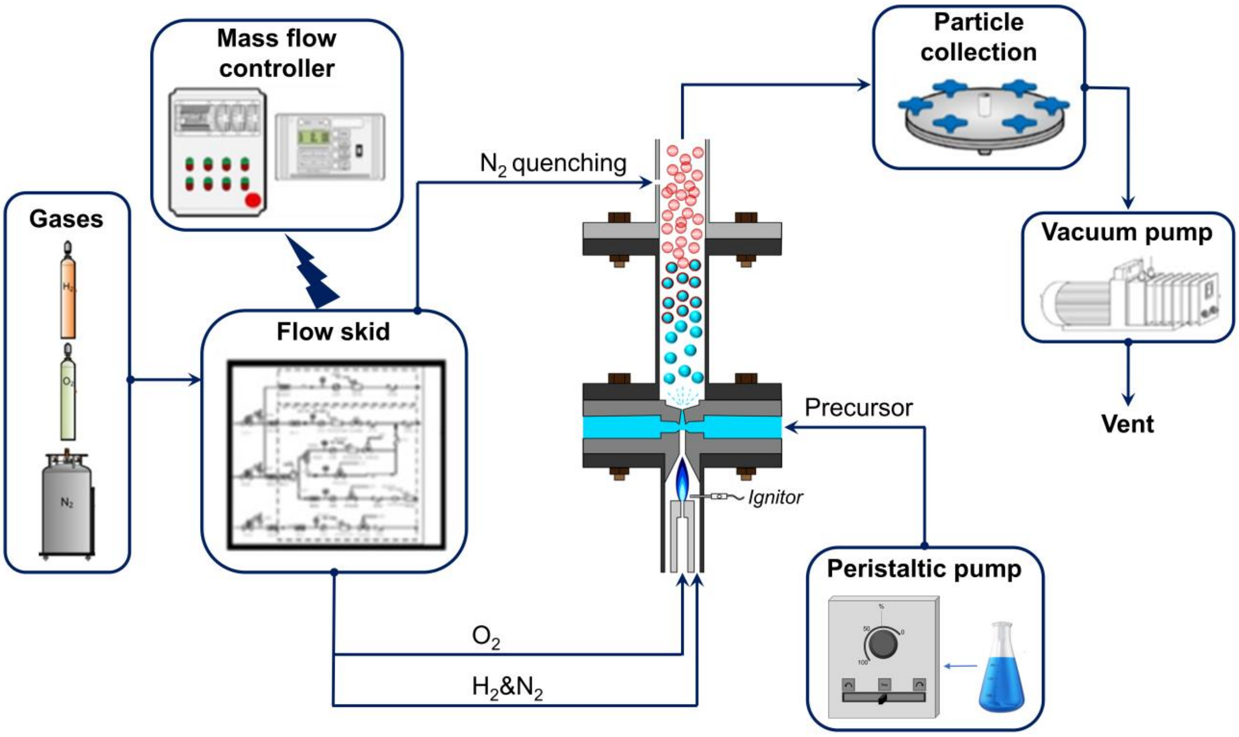

Supplementary Fig. 2. Schematic of the modified flame reactor.

The modified flame aerosol reactor retains the one-step, continuous synthesis approach of conventional flame spray pyrolysis technology,[1] but separates the flame and particle formation process into different regions, which results in a lower reaction temperature for particle formation. In this case, the materials form in a droplet-to-particle route rather than a gas-to-particle route that is often observed in conventional flame spray pyrolysis processes. Moreover, it allows use of aqueous inorganic salt solutions instead of metal organic precursors dissolved in flammable organic solvents, which greatly increases the availability of precursors for virtually all elements and decreases production cost and environmental impacts. In addition, it allows control of the particle formation environment to achieve either an oxidizing or reducing reaction atmosphere by controlling the flow rates of H_2 and O_2. The fast N_2 quenching downstream can prevent phase separation and particle sintering.

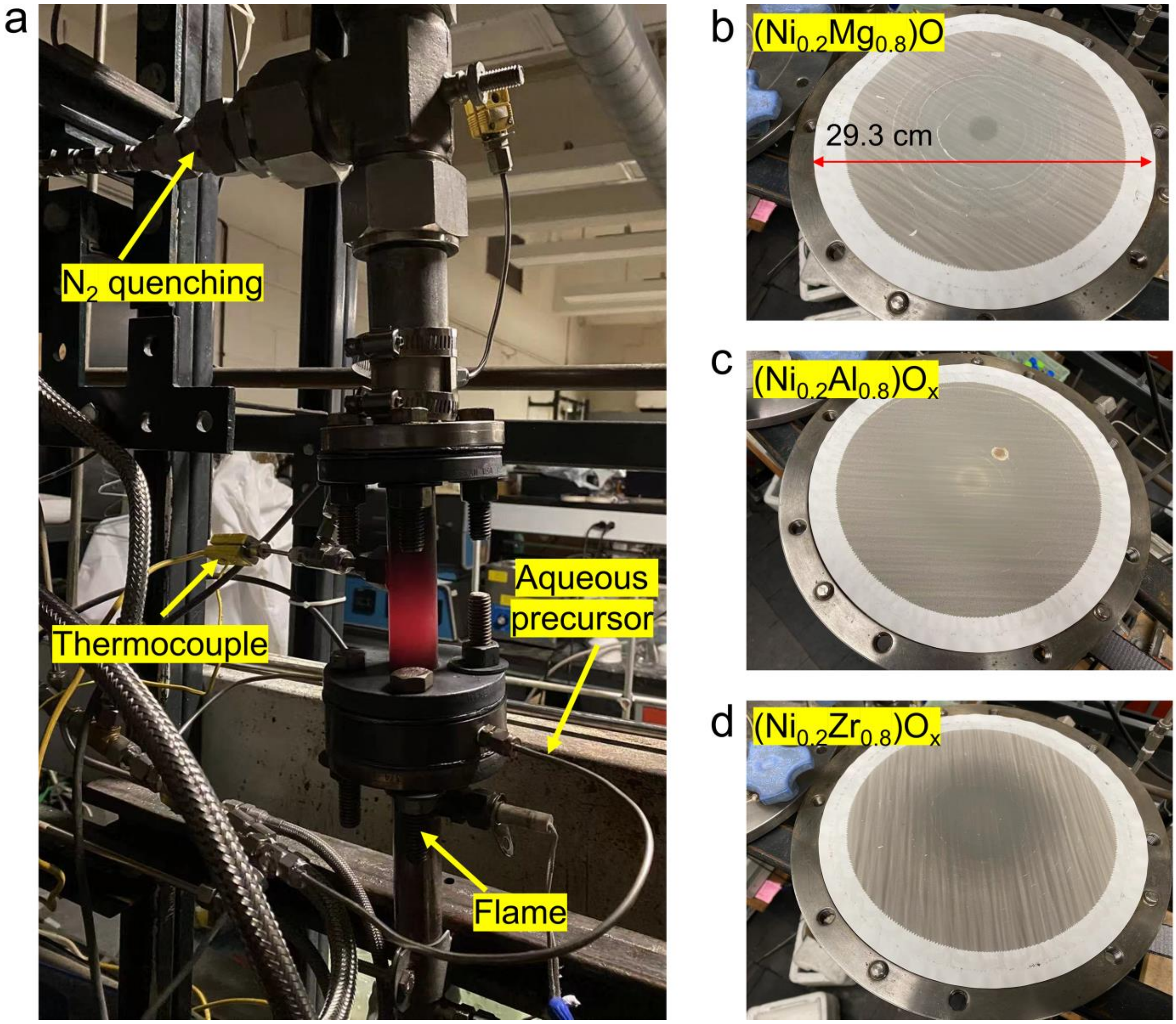

Supplementary Fig. 3. Photographs of **a.** flame reactor in operation, and typical **b.** $(Ni_{0.2}Mg_{0.8})O$, **c.** $(Ni_{0.2}Al_{0.8})O_x$, and **d.** $(Ni_{0.2}Zr_{0.8})O_x$ solid solution materials collected on a membrane filter.

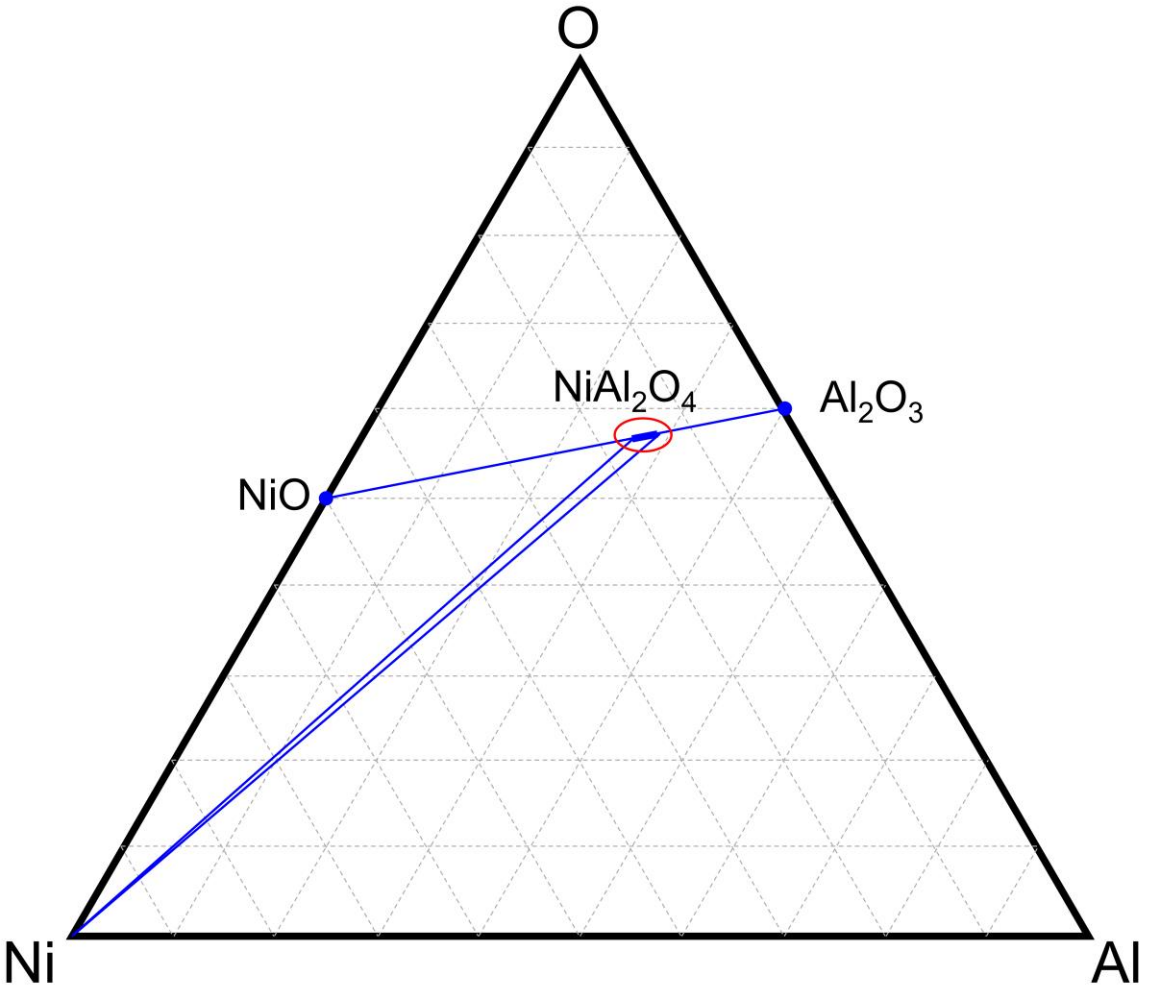

Supplementary Fig. 4. Isothermal section of Ni-Al-O ternary phase diagram at 1000 ºC,[2] showing a small miscible region near the $NiAl_2O_4$ compound stoichiometry.

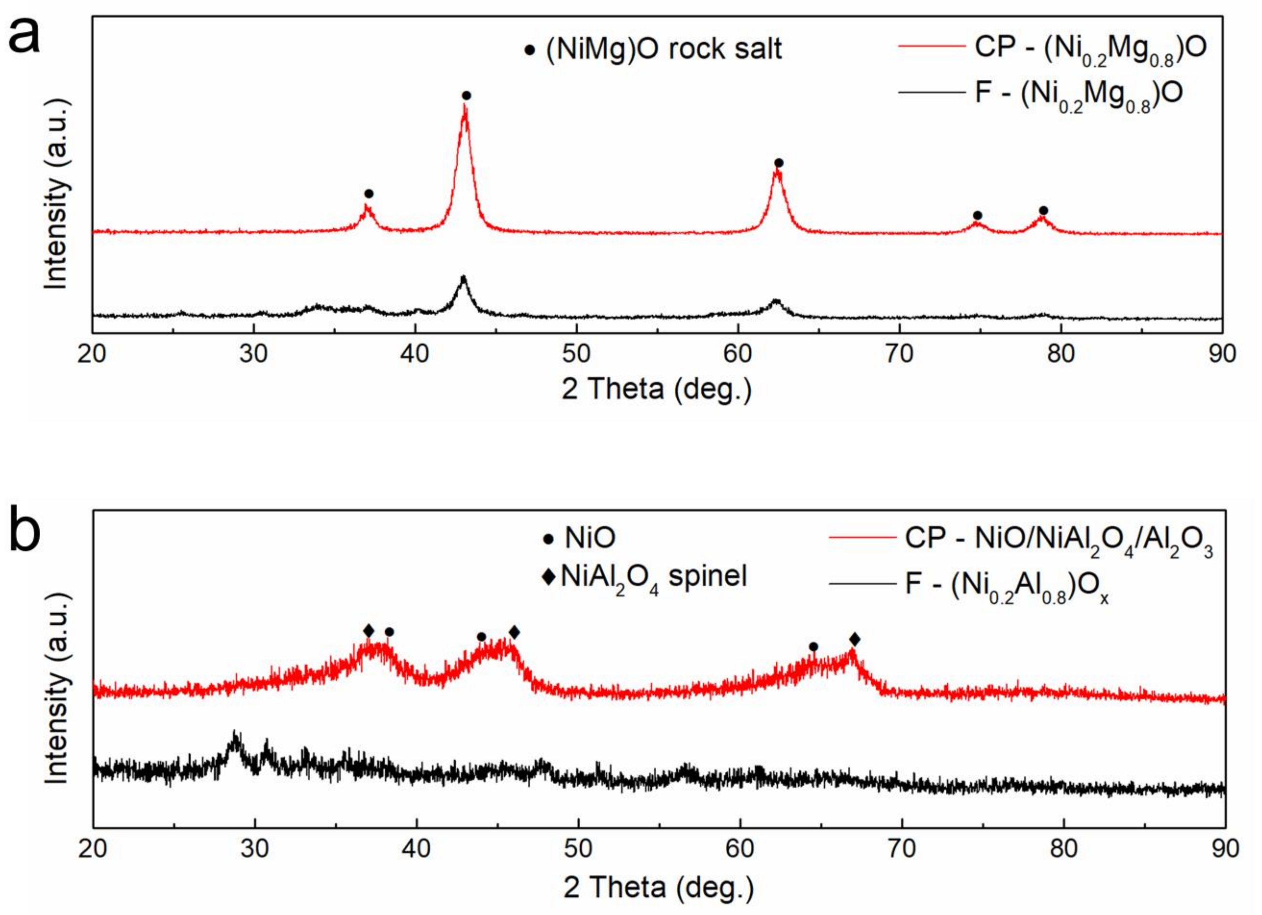

Supplementary Fig. 5. XRD patterns of **a.** CP-(NiMg)O and F-(NiMg)O; and **b.** CP-(NiAl)O_x and F-(NiAl)O_x.

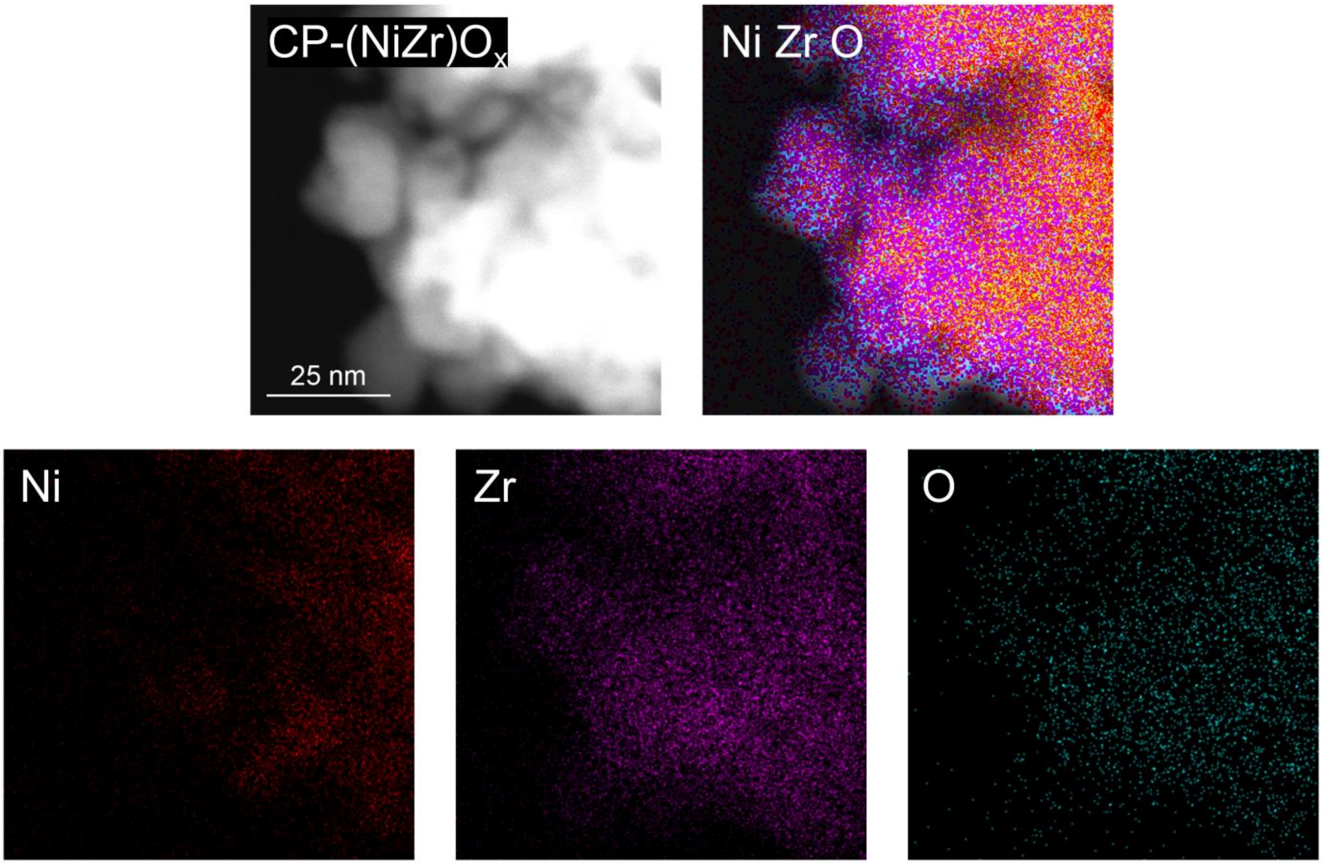

Supplementary Fig. 6. HAADF-STEM image and elemental maps, as labeled, of CP-(NiZr)O_x.

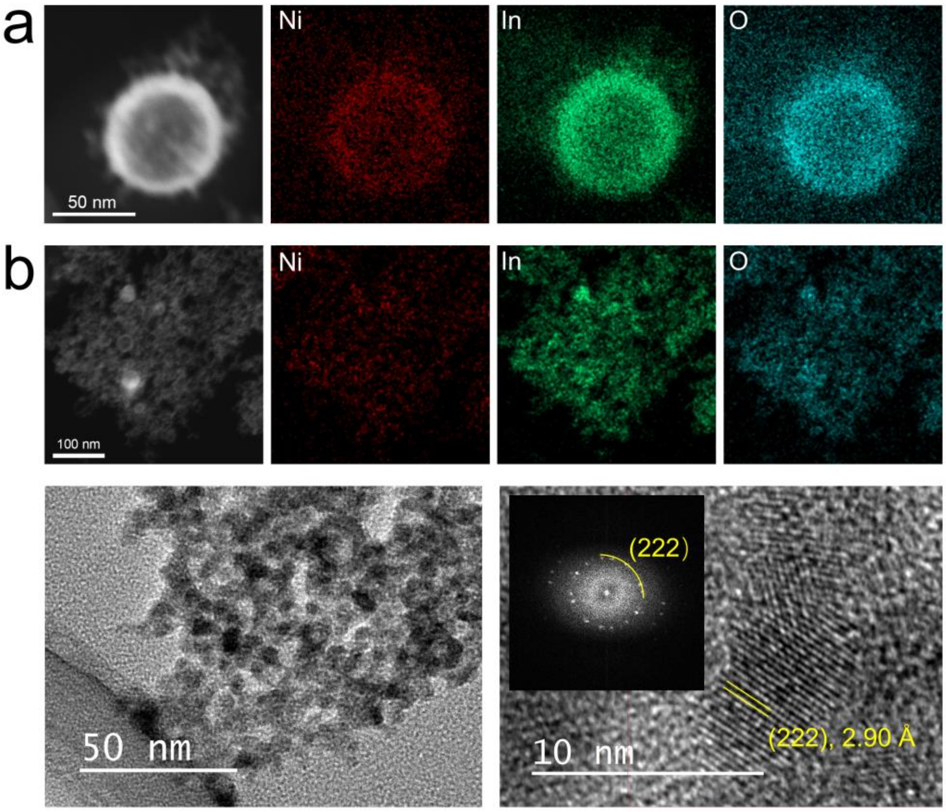

Supplementary Fig. 7. a. $(Ni_{0.1}In_{0.9})O_x$ nanoshell formed by a droplet-to-particle route; **b.** $(Ni_{0.1}In_{0.9})O_x$ nanoparticles formed by a gas-to-particle route, as for the nanoparticles on the surface of the nanoshell in Supplementary Fig. 9j. The two morphologies were observed in the same sample.

Two particle formation mechanisms are common in flame aerosol processes. These are droplet-to-particle and gas-to-particle routes, which depends on many parameters such as reactor configuration, reaction temperature, precursor species and concentration, and droplet size distribution. In the gas-to-particle route, the precursors or their decomposition products evaporate fully, and subsequent product nucleation and growth processes happens in the gas phase, finally forming nanoparticles.[3] In the droplet-to-particle route, each droplet is converted to a single, often hollow, solid particle by solvent evaporation and condensed phase reactions. In the current flame synthesis conditions, both routes simultaneously for $(Ni_{0.1}In_{0.9})O_x$ material. No separate NiO phase was observed in the XRD pattern of $(Ni_{0.1}In_{0.9})O_x$ (Supplementary Fig. 11j), indicating no phase separation in either nanoshell or nanoparticle morphologies. Furthermore, HAADF-STEM elemental mapping also showed the uniform distribution of Ni, In, and O in both nanoshell (Supplementary Fig. 7a) and nanoparticles (Supplementary Fig. 7b), and the single crystal structure of the nanoparticles was confirmed by HRTEM with FFT. This demonstrated that both droplet-to-particle and gas-to-particle routes form uniform ceramic solid solutions in the current flame synthesis method.

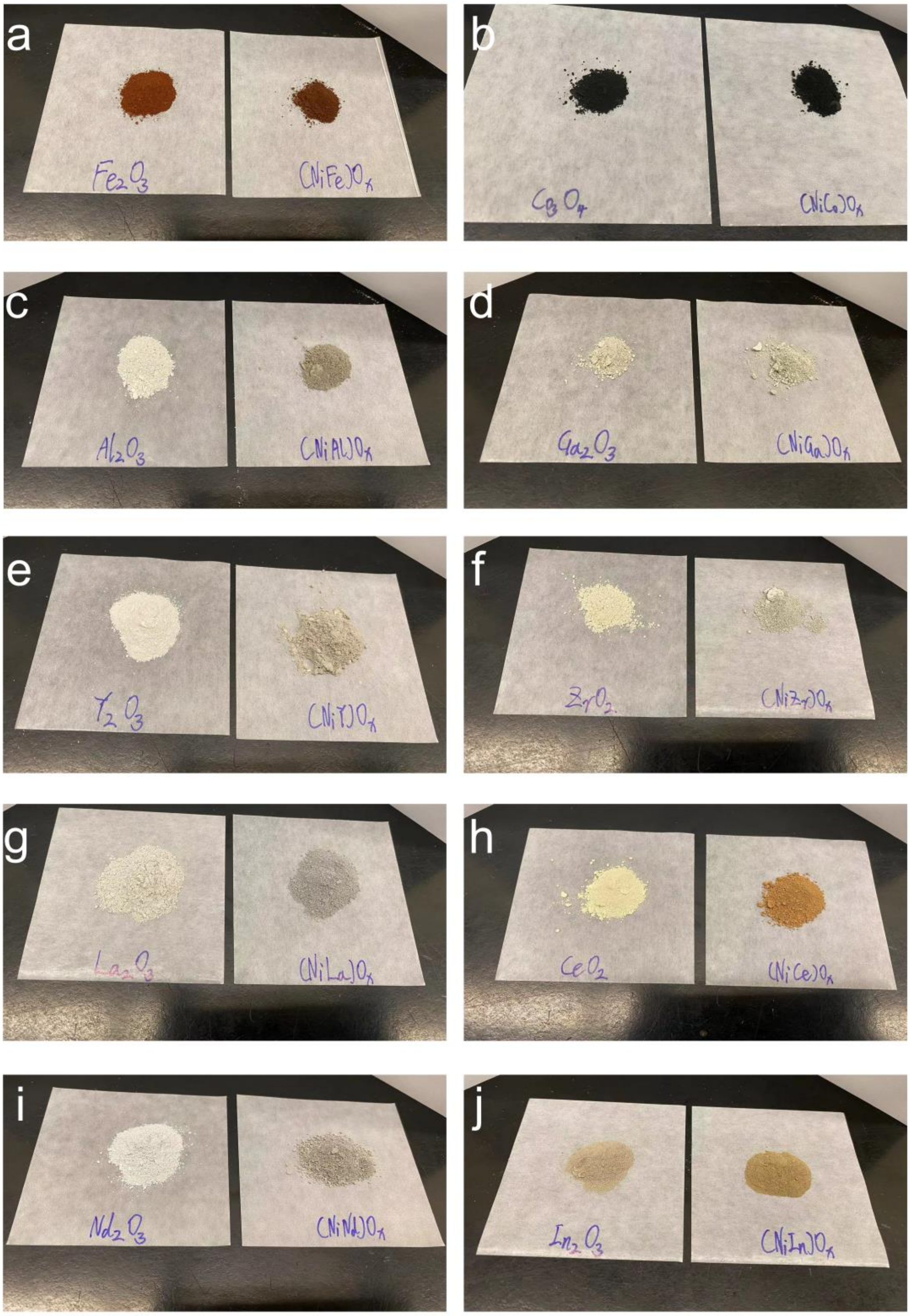

Supplementary Fig. 8. Photographs of flame synthesized single metal oxides and Ni-containing binary ceramic solid solutions. **a.** Fe_2O_3 and $(Ni_{0.1}Fe_{0.9})O_x$; **b.** Co_3O_4 and $(Ni_{0.1}Co_{0.9})O_x$; **c.** Al_2O_3 and $(Ni_{0.1}Al_{0.9})O_x$; **d.** Ga_2O_3 and $(Ni_{0.1}Ga_{0.9})O_x$; **e.** Y_2O_3 and $(Ni_{0.1}Y_{0.9})O_x$; **f.** ZrO_2 and $(Ni_{0.1}Zr_{0.9})O_x$; **g.** La_2O_3 and $(Ni_{0.1}La_{0.9})O_x$; **h.** CeO_2 and $(Ni_{0.1}Ce_{0.9})O_x$; **i.** Nd_2O_3 and $(Ni_{0.1}Nd_{0.9})O_x$and **j.** In_2O_3 $(Ni_{0.1}In_{0.9})O_x$**.** The oxides showed a visible color change upon NiO incorporation.

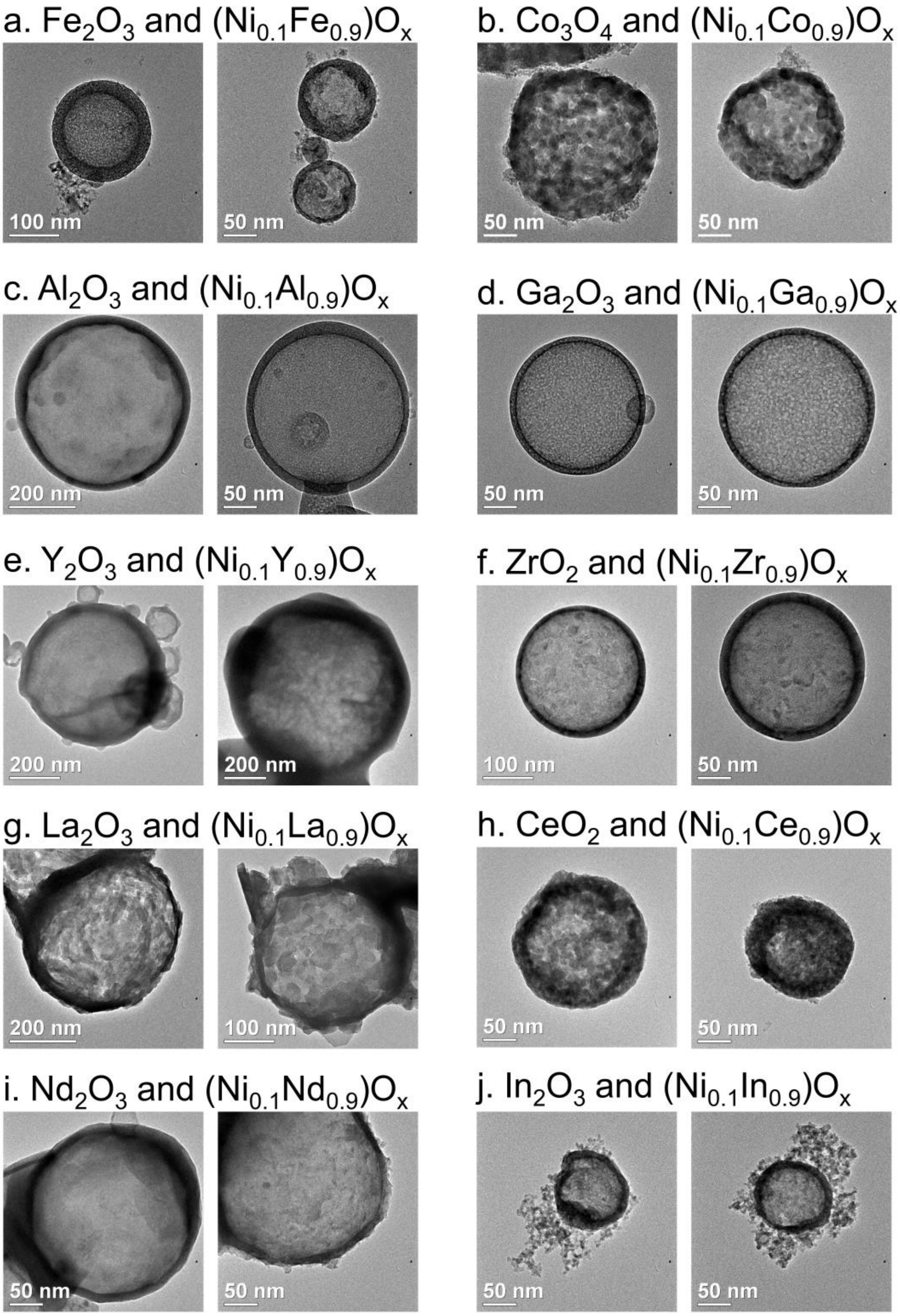

Supplementary Fig. 9. Representative TEM images of flame synthesized single metal oxides and Ni-containing binary ceramic solid solutions. **a.** Fe_2O_3 and $(Ni_{0.1}Fe_{0.9})O_x$; **b.** Co_3O_4 and $(Ni_{0.1}Co_{0.9})O_x$; **c.** Al_2O_3 and $(Ni_{0.1}Al_{0.9})O_x$; **d.** Ga_2O_3 and $(Ni_{0.1}Ga_{0.9})O_x$; **e.** Y_2O_3 and $(Ni_{0.1}Y_{0.9})O_x$; **f.** ZrO_2 and $(Ni_{0.1}Zr_{0.9})O_x$; **g.** La_2O_3 and $(Ni_{0.1}La_{0.9})O_x$; **h.** CeO_2 and $(Ni_{0.1}Ce_{0.9})O_x$; **i.** Nd_2O_3 and $(Ni_{0.1}Nd_{0.9})O_x$ and **j.** In_2O_3 $(Ni_{0.1}In_{0.9})O_x$. The nanoshell morphology was retained upon Ni incorporation.

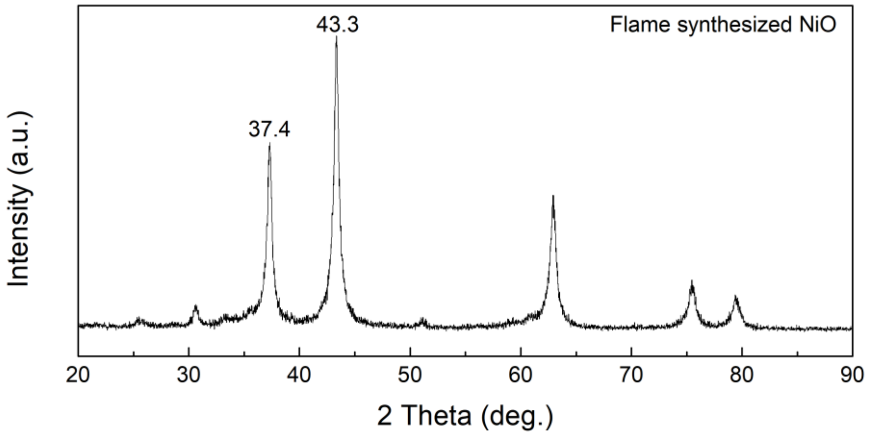

Supplementary Fig. 10. XRD pattern of flame synthesized NiO. The (111) and (200) planes are shown at 2θ of 37.4º and 43.3º.

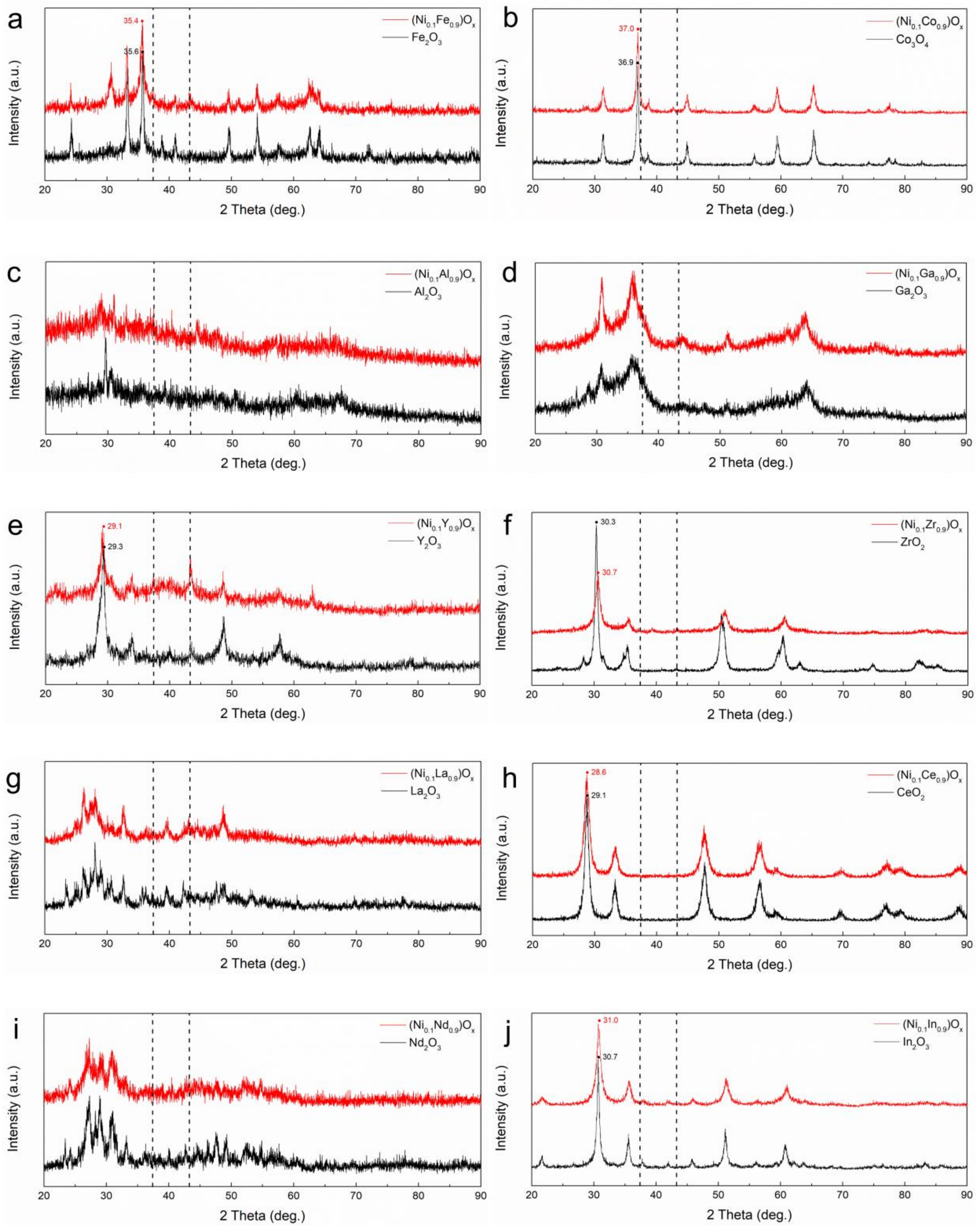

Supplementary Fig. 11. XRD patterns of flame synthesized single metal oxides and Ni-containing binary ceramic solid solutions. **a.** Fe_2O_3 and $(Ni_{0.1}Fe_{0.9})O_x$; **b.** Co_3O_4 and $(Ni_{0.1}Co_{0.9})O_x$; **c.** Al_2O_3 and $(Ni_{0.1}Al_{0.9})O_x$; **d.** Ga_2O_3 and $(Ni_{0.1}Ga_{0.9})O_x$; **e.** Y_2O_3 and $(Ni_{0.1}Y_{0.9})O_x$; **f.** ZrO_2 and $(Ni_{0.1}Zr_{0.9})O_x$; **g.** La_2O_3 and $(Ni_{0.1}La_{0.9})O_x$; **h.** CeO_2 and $(Ni_{0.1}Ce_{0.9})O_x$; **i.** Nd_2O_3 and $(Ni_{0.1}Nd_{0.9})O_x$and **j.** In_2O_3 $(Ni_{0.1}In_{0.9})O_x$ materials, demonstrating the homogeneous mixing of immiscible. Compared with pure solvent metal oxides, the peak shifts in XRD was due to the change in lattice parameters after Ni doping.

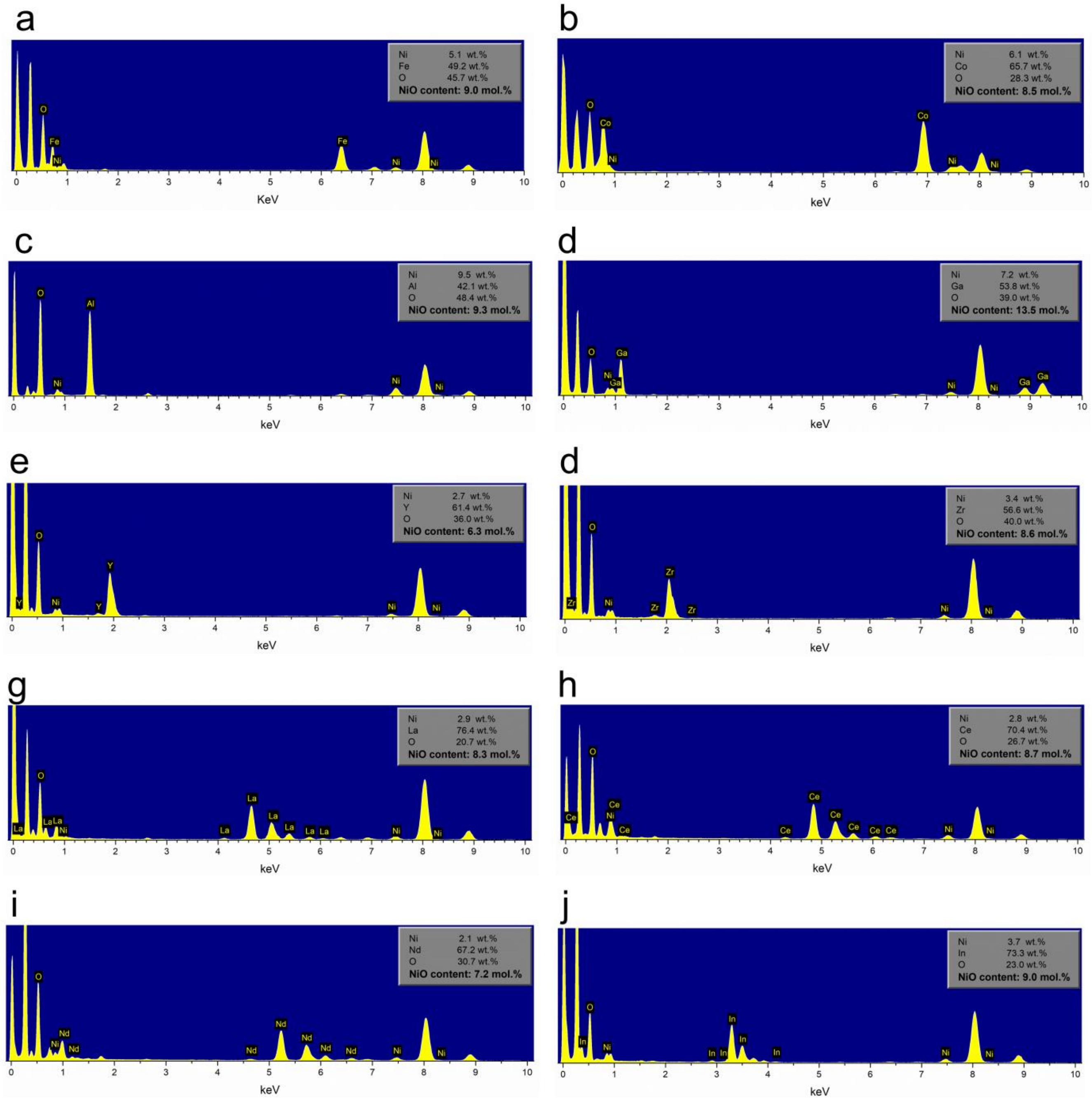

Supplementary Fig. 12. EDS spectra of flame synthesized Ni-containing binary ceramic solid solutions corresponding to the elemental maps in Fig. 3c-k and Supplementary Fig. S7a. **a.** $(Ni_{0.1}Fe_{0.9})O_x$; **b.** $(Ni_{0.1}Co_{0.9})O_x$; **c.** $(Ni_{0.1}Al_{0.9})O_x$; **d.** $(Ni_{0.1}Ga_{0.9})O_x$; **e.** $(Ni_{0.1}Y_{0.9})O_x$; **f.** $(Ni_{0.1}Zr_{0.9})O_x$; **g.** $(Ni_{0.1}La_{0.9})O_x$; **h.** $(Ni_{0.1}Ce_{0.9})O_x$; **i.** $(Ni_{0.1}Nd_{0.9})O_x$ and **j.** $(Ni_{0.1}In_{0.9})O_x$. The small deviations from the molar ratio provided in the precursor solution (10 mol.%) can be attributed to differences in vapor pressure of precursor salts, which is a common feature in non-equilibrium synthesis methods. A desired final composition can be achieved by corresponding small changes in the precursor composition.[4]

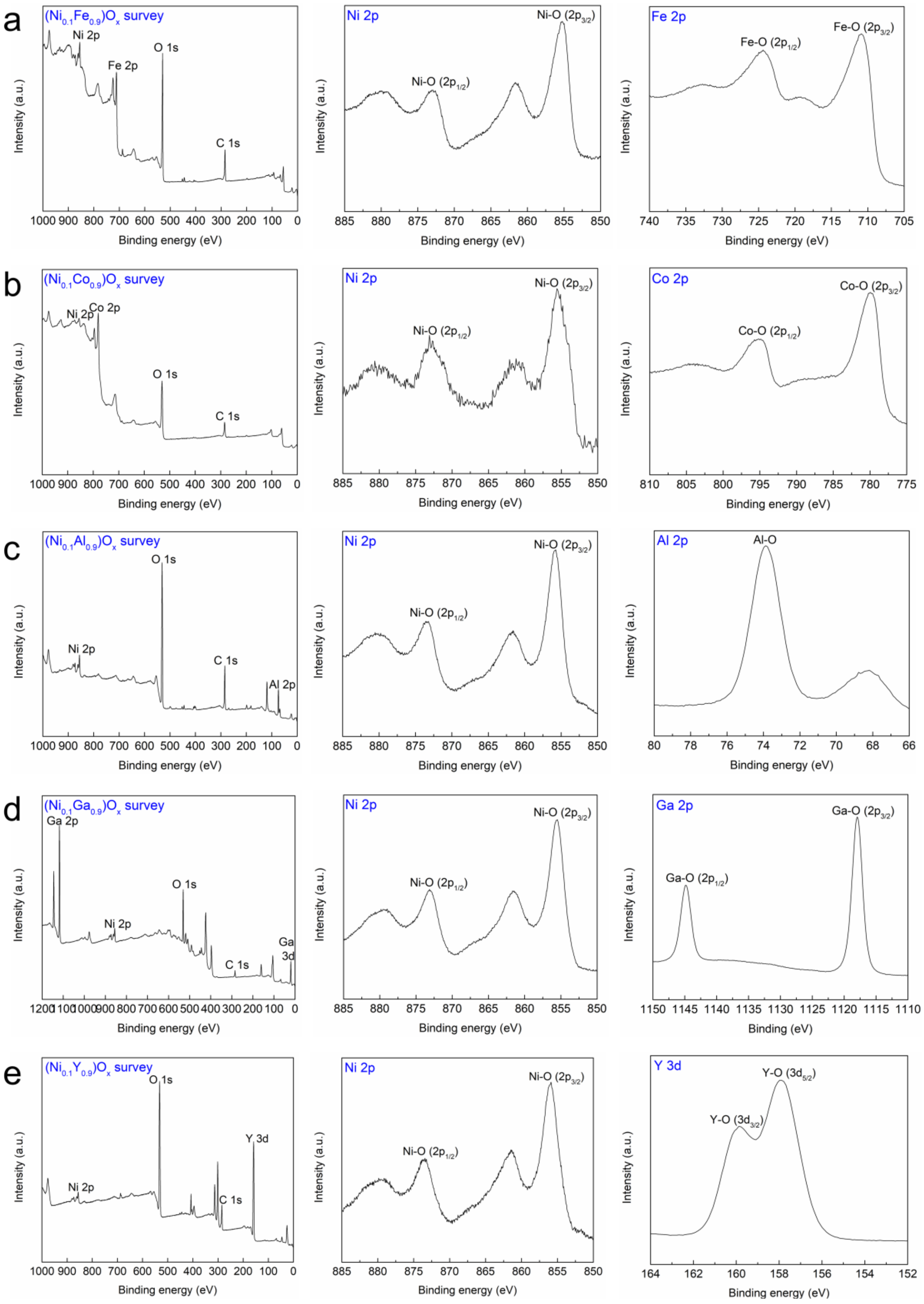

Supplementary Fig. 13. XPS spectra of flame synthesized Ni-containing binary ceramic solid solutions. **a.** $(Ni_{0.1}Fe_{0.9})O_x$; **b.** $(Ni_{0.1}Co_{0.9})O_x$; **c.** $(Ni_{0.1}Al_{0.9})O_x$; **d.** $(Ni_{0.1}Ga_{0.9})O_x$; **e.** $(Ni_{0.1}Y_{0.9})O_x$, confirming the cation state of all metal elements in these ceramic solid solution materials.

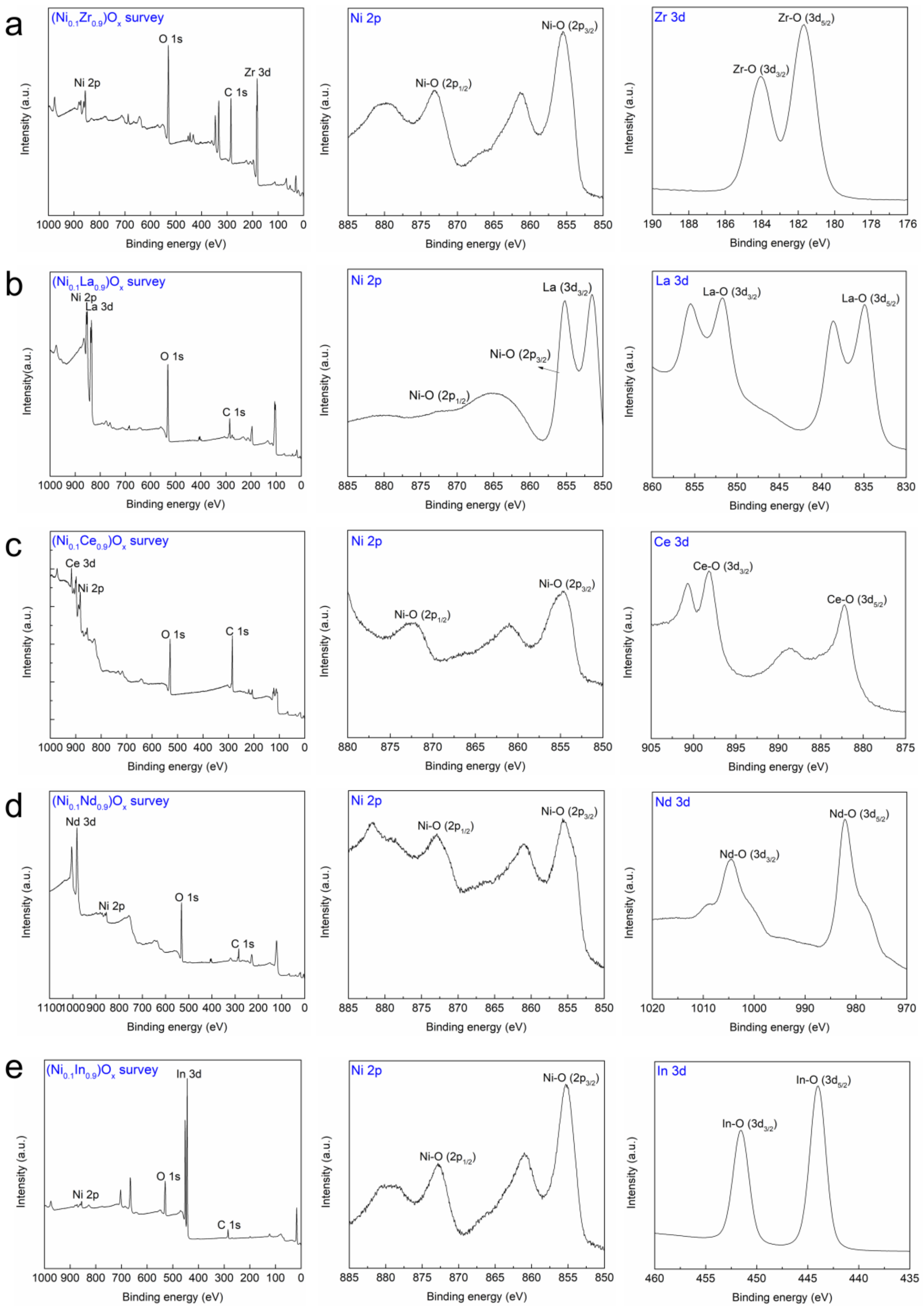

Supplementary Fig. 14. XPS spectra of flame synthesized Ni-containing binary ceramic solid solutions. **a.** $(Ni_{0.1}Zr_{0.9})O_x$; **b.** $(Ni_{0.1}La_{0.9})O_x$; **c.** $(Ni_{0.1}Ce_{0.9})O_x$; **d.** $(Ni_{0.1}Nd_{0.9})O_x$; **e.** $(Ni_{0.1}In_{0.9})O_x$, confirming the cation state of all metal elements in these ceramic solid solution materials.

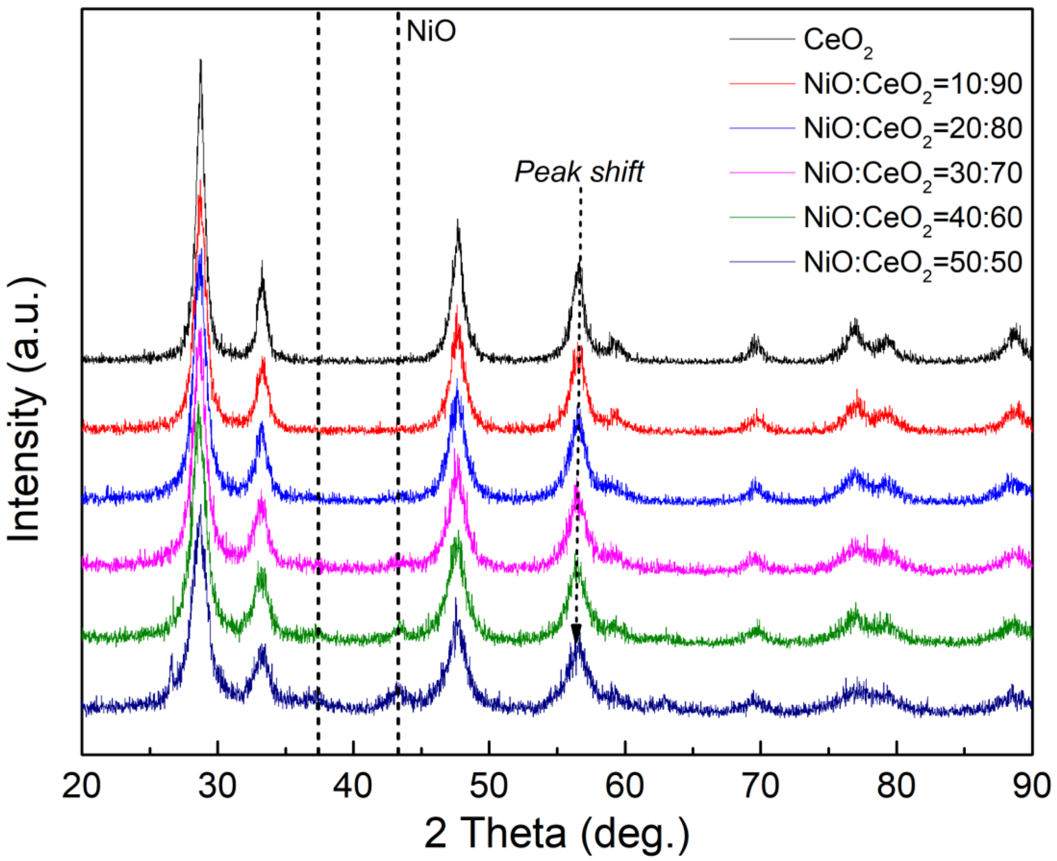

Supplementary Fig. 15. XRD patterns of $(NiCe)O_x$ solid solution materials with increased Ni content from 0 to 50 mol.%.

To further probe the limits of this method of mixing immiscible elements, we selected $(NiCe)O_x$ solid solution as an example and increased the Ni content from 0 to 50 mol.% in the precursor. Even at equimolar content of Ni and Ce (1:1), the XRD still showed primarily the fluorite phase of CeO_2, with very small NiO peaks that were not present for lower Ni content.

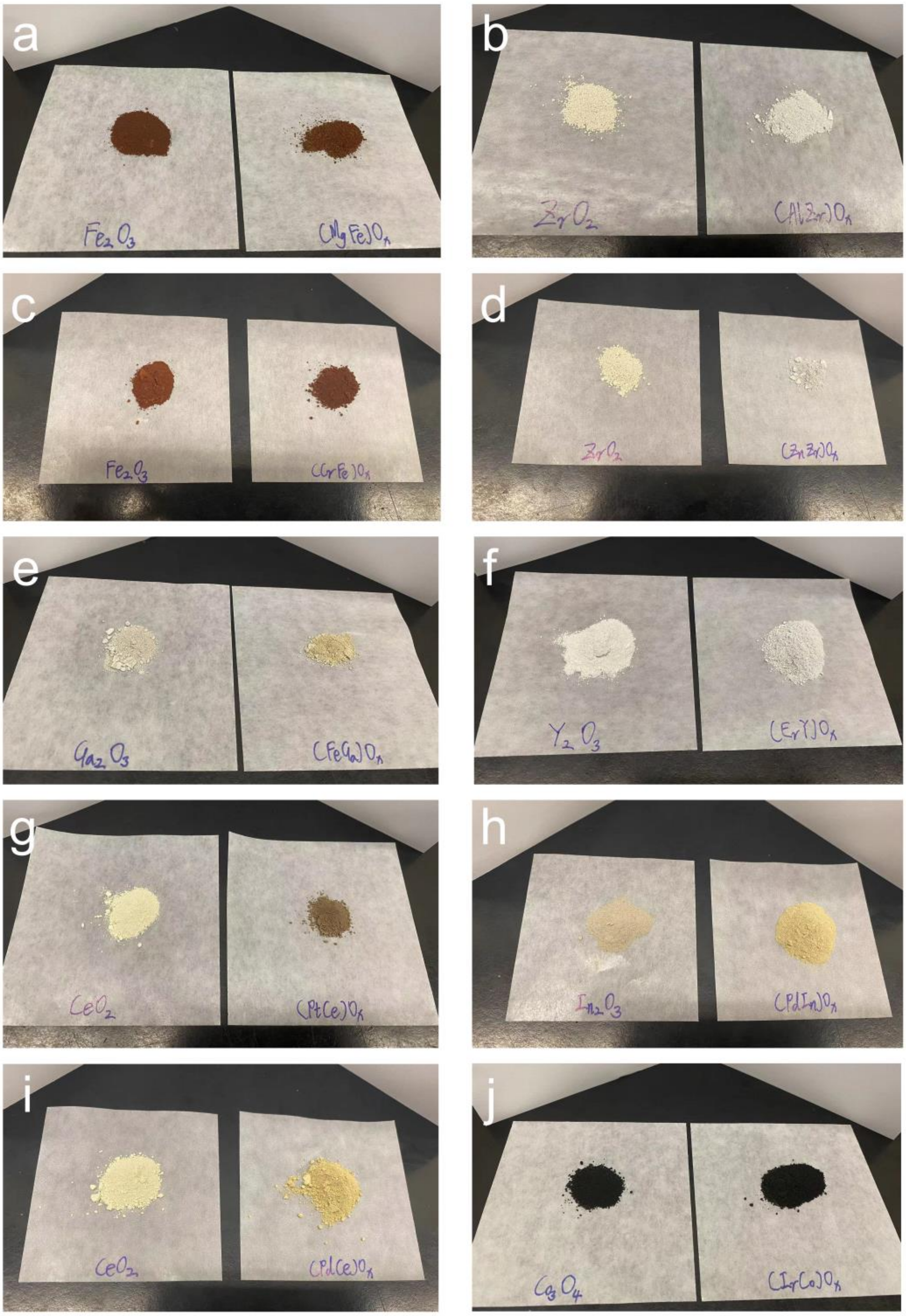

Supplementary Fig. 16. Photographs of flame synthesized single metal oxides and corresponding binary ceramic solid solutions. **a.** Fe_2O_3 and $(Mg_{0.1}Fe_{0.9})O_x$; **b.** ZrO_2 and $(Al_{0.1}Zr_{0.9})O_x$; **c.** Fe_2O_3 and $(Cr_{0.1}Fe_{0.9})O_x$; **d.** ZrO_2 and $(Zn_{0.1}Zr_{0.9})O_x$; **e.** Ga_2O_3 and $(Fe_{0.1}Ga_{0.9})O_x$; **f.** Y_2O_3 and $(Er_{0.1}Y_{0.9})O_x$; **g.** CeO_2 and $(Pt_{0.02}Ce_{0.98})O_x$; **h.** In_2O_3 and $(Pd_{0.02}In_{0.98})O_x$; **i.** CeO_2 and $(Pd_{0.02}Ce_{0.98})O_x$ and **j.** Co_3O_4 and $(Ir_{0.02}Co_{0.98})O_x$.

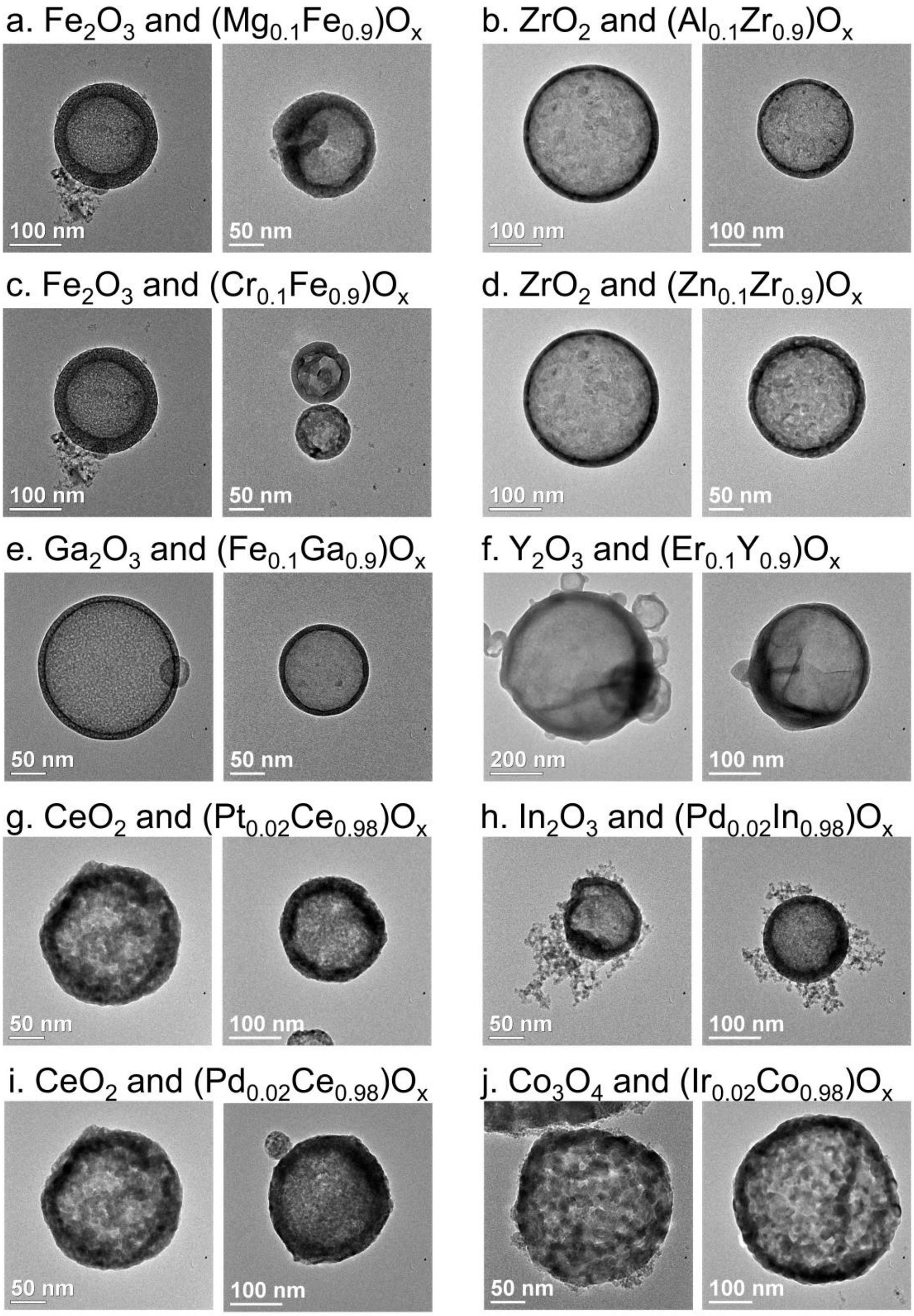

Supplementary Fig. 17. TEM images of flame synthesized single metal oxides and corresponding binary ceramic solid solutions. **a.** Fe_2O_3 and $(Mg_{0.1}Fe_{0.9})O_x$; **b.** ZrO_2 and $(Al_{0.1}Zr_{0.9})O_x$; **c.** Fe_2O_3 and $(Cr_{0.1}Fe_{0.9})O_x$; **d.** ZrO_2 and $(Zn_{0.1}Zr_{0.9})O_x$; **e.** Ga_2O_3 and $(Fe_{0.1}Ga_{0.9})O_x$; **f.** Y_2O_3 and $(Er_{0.1}Y_{0.9})O_x$; **g.** CeO_2 and $(Pt_{0.02}Ce_{0.98})O_x$; **h.** In_2O_3 and $(Pd_{0.02}In_{0.98})O_x$; **i.** CeO_2 and $(Pd_{0.02}Ce_{0.98})O_x$ and **j.** Co_3O_4 and $(Ir_{0.02}Co_{0.98})O_x$.

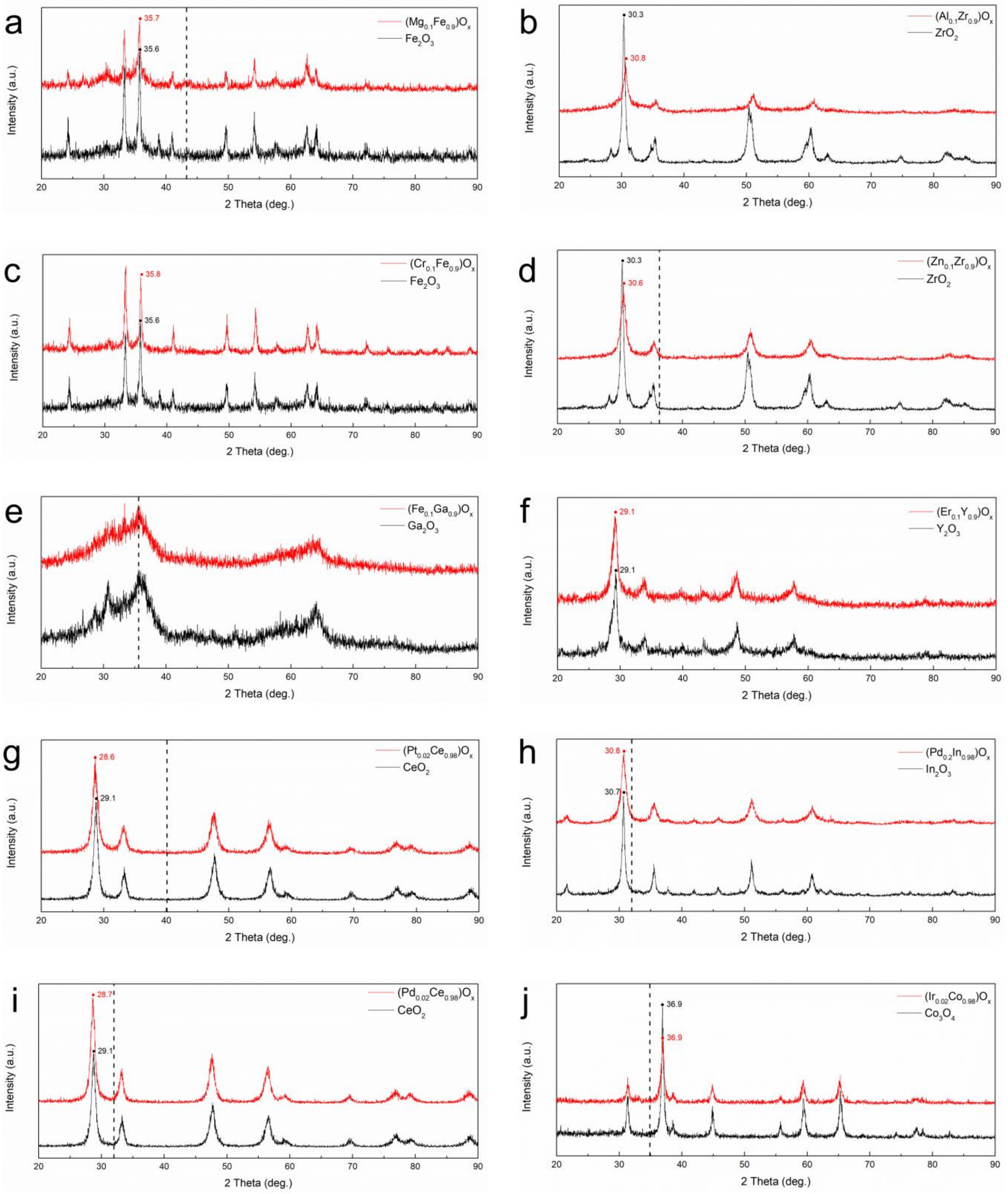

Supplementary Fig. 18. XRD patterns of the flame synthesized single metal oxides and corresponding binary ceramic solid solutions. **a.** Fe_2O_3 and $(Mg_{0.1}Fe_{0.9})O_x$; **b.** ZrO_2 and $(Al_{0.1}Zr_{0.9})O_x$; **c.** Fe_2O_3 and $(Cr_{0.1}Fe_{0.9})O_x$; **d.** ZrO_2 and $(Zn_{0.1}Zr_{0.9})O_x$; **e.** Ga_2O_3 and $(Fe_{0.1}Ga_{0.9})O_x$; **f.** Y_2O_3 and $(Er_{0.1}Y_{0.9})O_x$; **g.** CeO_2 and $(Pt_{0.02}Ce_{0.98})O_x$; **h.** In_2O_3 and $(Pd_{0.02}In_{0.98})O_x$; **i.** CeO_2 and $(Pd_{0.02}Ce_{0.98})O_x$ and **j.** Co_3O_4 and $(Ir_{0.02}Co_{0.98})O_x$.

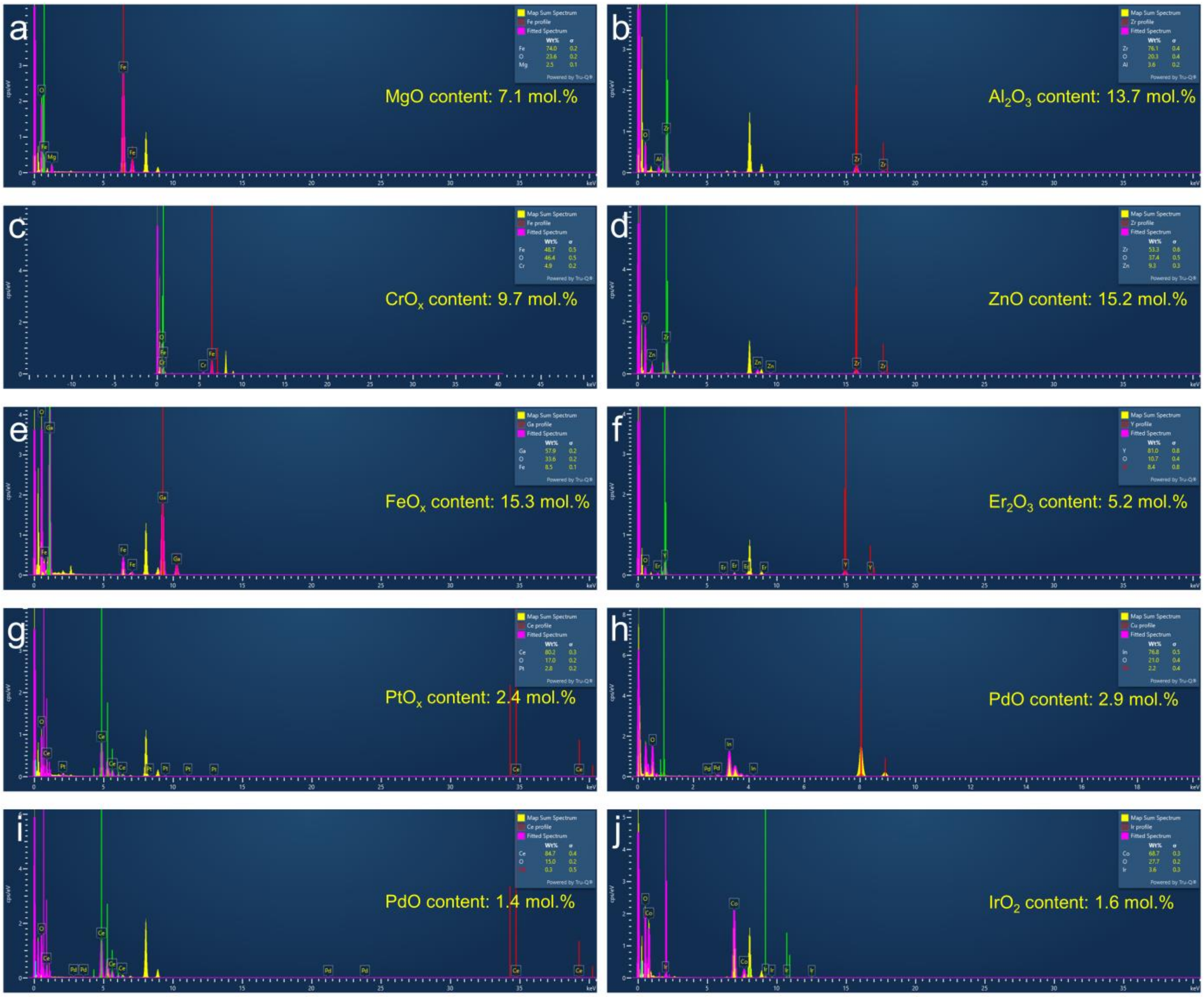

Supplementary Fig. 19. EDS spectra of flame synthesized binary ceramic solid solutions corresponding to the elemental maps in Fig. 4. **a.** $(Mg_{0.1}Fe_{0.9})O_x$; **b.** $(Al_{0.1}Zr_{0.9})O_x$; **c.** $(Cr_{0.1}Fe_{0.9})O_x$; **d.** $(Zn_{0.1}Zr_{0.9})O_x$; **e.** $(Fe_{0.1}Ga_{0.9})O_x$; **f.** $(Er_{0.1}Y_{0.9})O_x$; **g.** $(Pt_{0.02}Ce_{0.98})O_x$; **h.** $(Pd_{0.02}In_{0.98})O_x$; **i.** $(Pd_{0.02}Ce_{0.98})O_x$ and **j.** $(Ir_{0.02}Co_{0.98})O_x$.

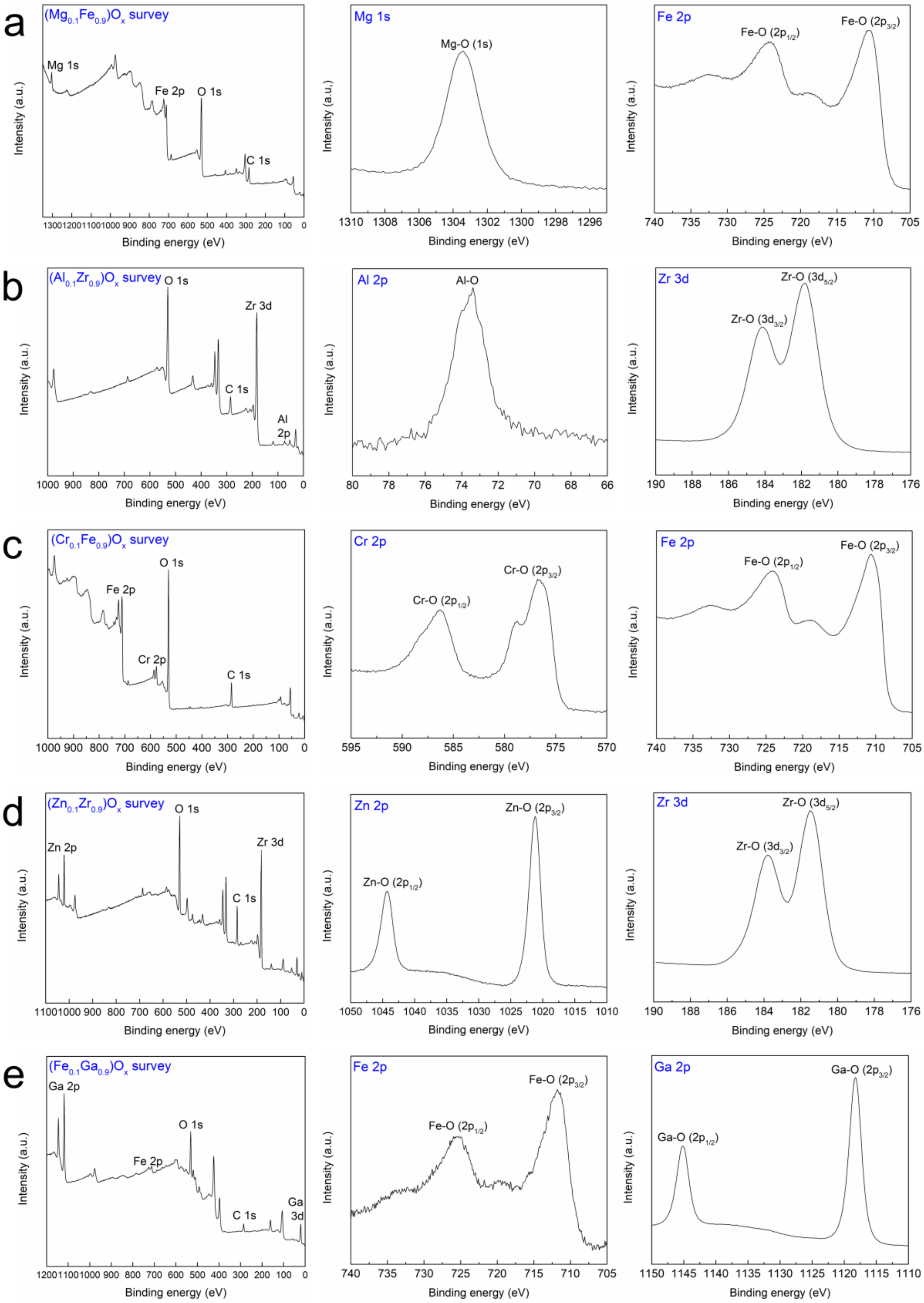

Supplementary Fig. 20. XPS spectra of flame synthesized binary ceramic solid solutions. **A.** $(Mg_{0.1}Fe_{0.9})O_x$; **B.** $(Al_{0.1}Zr_{0.9})O_x$; **C.** $(Cr_{0.1}Fe_{0.9})O_x$; **D.** $(Zn_{0.1}Zr_{0.9})O_x$ and **E.** $(Fe_{0.1}Ga_{0.9})O_x$.

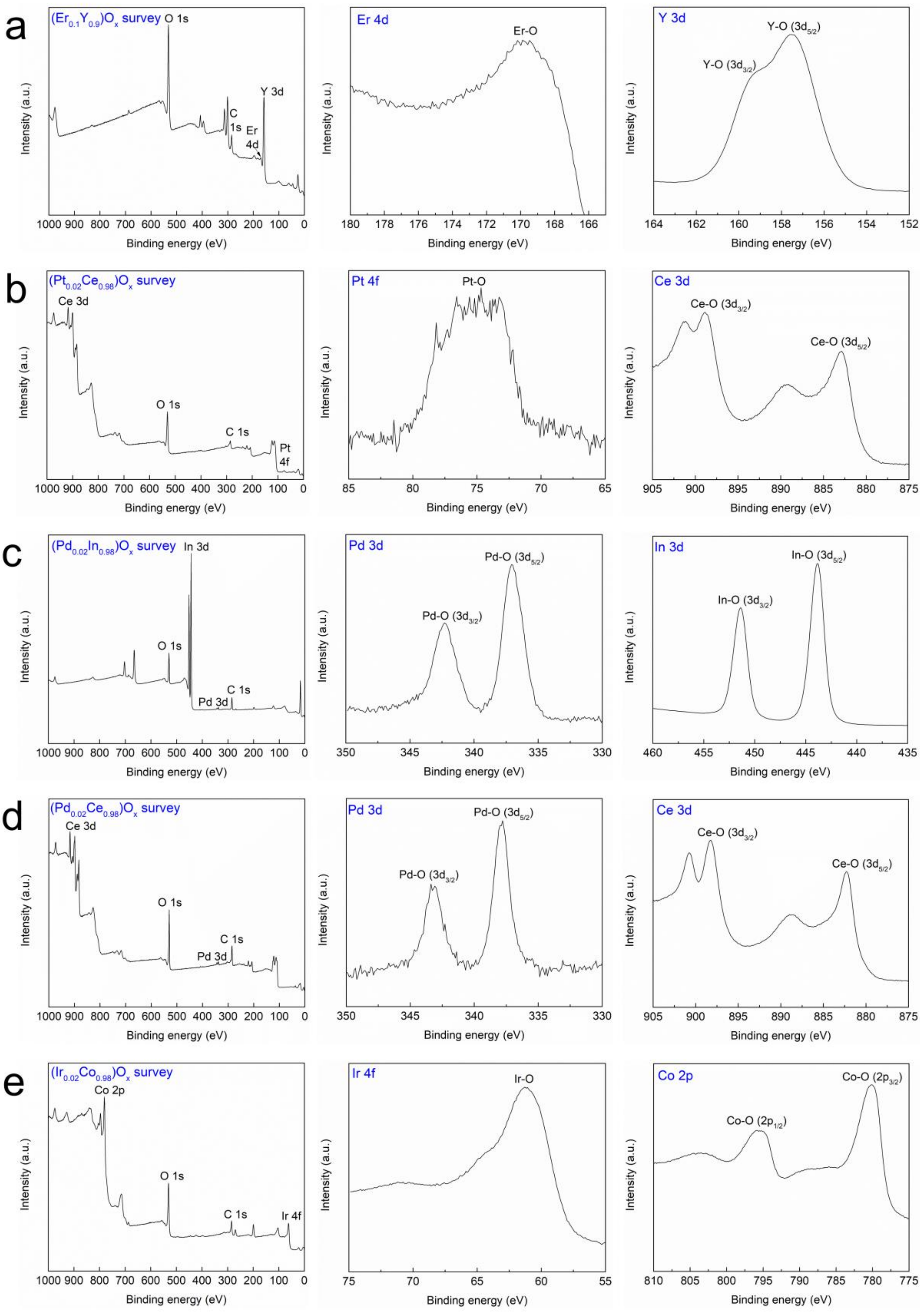

Supplementary Fig. 21. XPS spectra of flame synthesized binary ceramic solid solutions. **a.** $(Er_{0.1}Y_{0.9})O_x$; **b.** $(Pt_{0.02}Ce_{0.98})O_x$; **c.** $(Pd_{0.02}In_{0.98})O_x$; **d.** $(Pd_{0.02}Ce_{0.98})O_x$ and **e.** $(Ir_{0.02}Co_{0.98})O_x$.

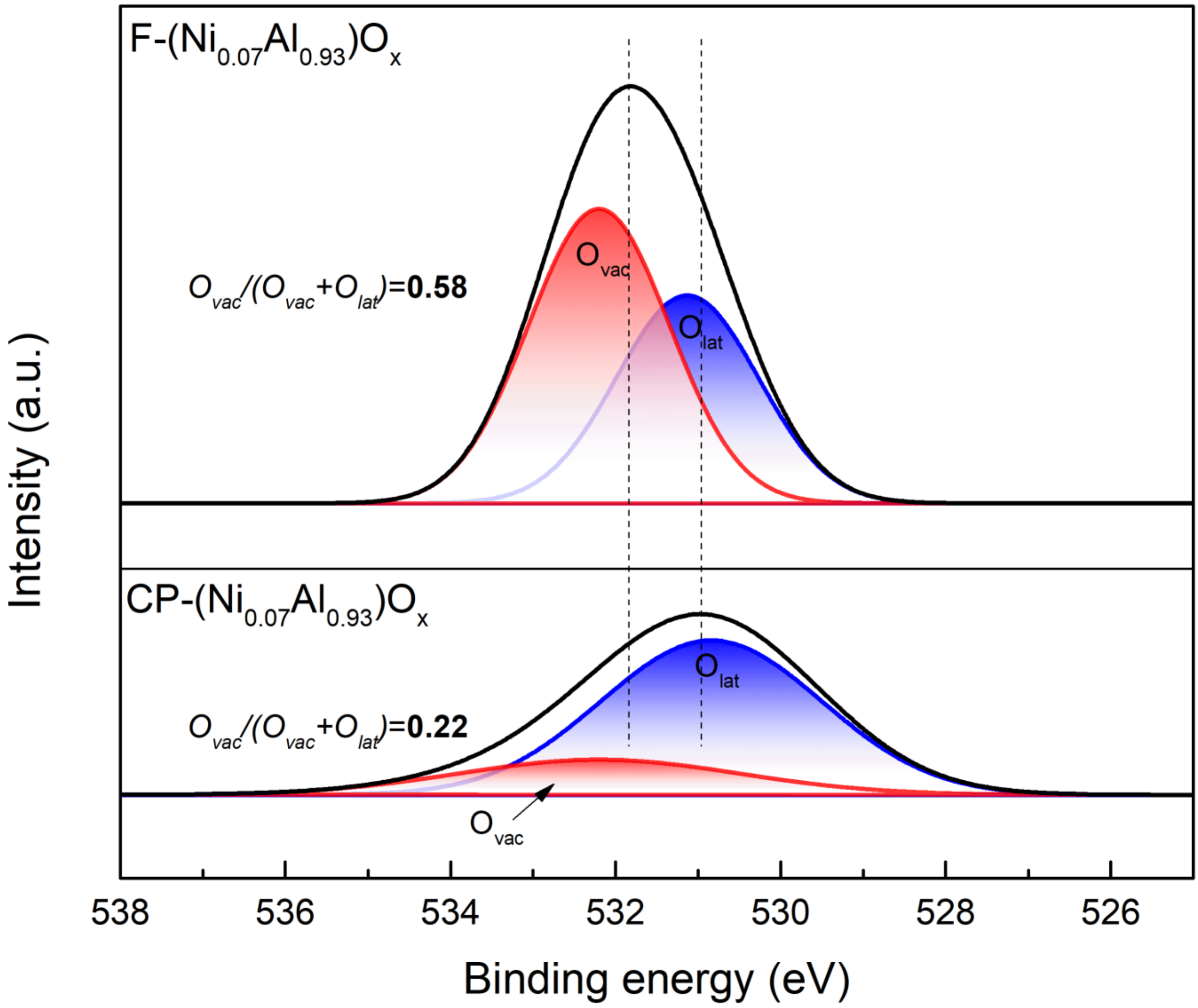

Supplementary Fig. 22. O *1s* XPS spectra of F-($Ni_{0.07}Al_{0.93}$)O_x and CP-($Ni_{0.07}Al_{0.93}$)O_x.

The surface oxygen vacancies of flame synthesized F-($Ni_{0.07}Al_{0.93}$)O_x and co-precipitation synthesized CP-($Ni_{0.07}Al_{0.93}$)O_x were characterized by O *1s* XPS spectra, where the peak at ~531 eV can be attributed to surface lattice oxygen, while the peak at ~532 eV can be attributed to the surface oxygen absorbed on oxygen vacancy, which reflect the amount of oxygen vacancy.[5] The results suggested that the oxygen vacancy concentration of F-($Ni_{0.07}Al_{0.93}$)O_x was much higher than CP-($Ni_{0.07}Al_{0.93}$)O_x, demonstrating that the rapid flame synthesis and exsolution generated more defects compared to the mild equilibrium synthesis process, and doping Ni atoms in Al_2O_3 lattice also favored lattice distortion and formation of oxygen vacancies.

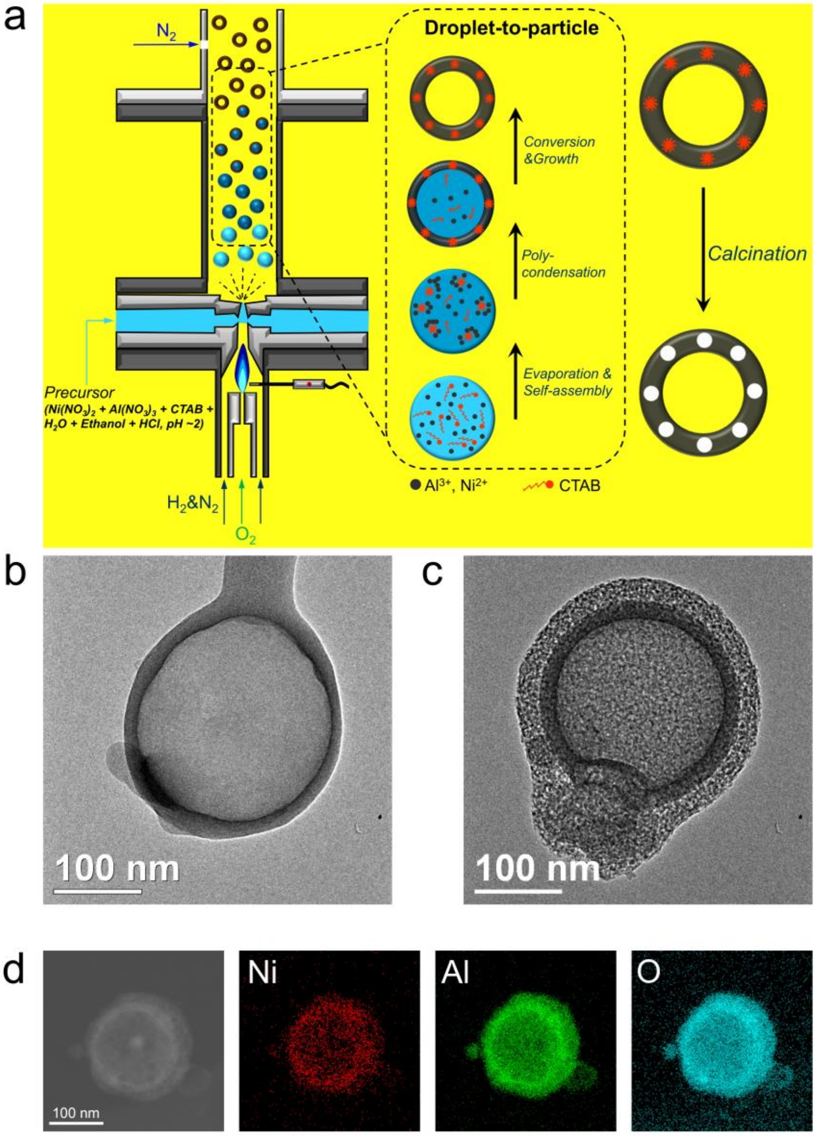

Supplementary Fig. 23. a. Schematic illustration of pore templating in $(Ni_{0.07}Al_{0.93})O_x$ nanoshell in the flame aerosol process; **b, c.** TEM images of $(Ni_{0.07}Al_{0.93})O_x$ solid solutions without and with template addition;**d.** HAADF-STEM image and elemental maps of the flame synthesized porous and hollow $(Ni_{0.07}Al_{0.93})O_x$ solid solution.

In this case, CTAB was added in to the $Ni(NO_3)_2$ and $Al(NO_3)_3$ precursor solution. Droplet evaporation increased the CTAB concentration and drove CTAB self-assembly into cylindrical micelles that template mesopores. Meanwhile, Ni^{2+} and Al^{3+} co-deposited on the micelle surfaces as concentration increased and finally formed a hybrid inorganic-organic intermediate product in the flame aerosol process. Removal of the micelle template by calcination (or washing with ethanol) generates mesopores (Supplementary Fig. 23a). TEM images confirmed the successful creation of dense pores compared with the non-porous $(Ni_{0.07}Al_{0.93})O_x$ produced without a templating agent (Supplementary Fig. 23b, c), while the distribution of the elements remained uniform (Supplementary Fig. 23d).

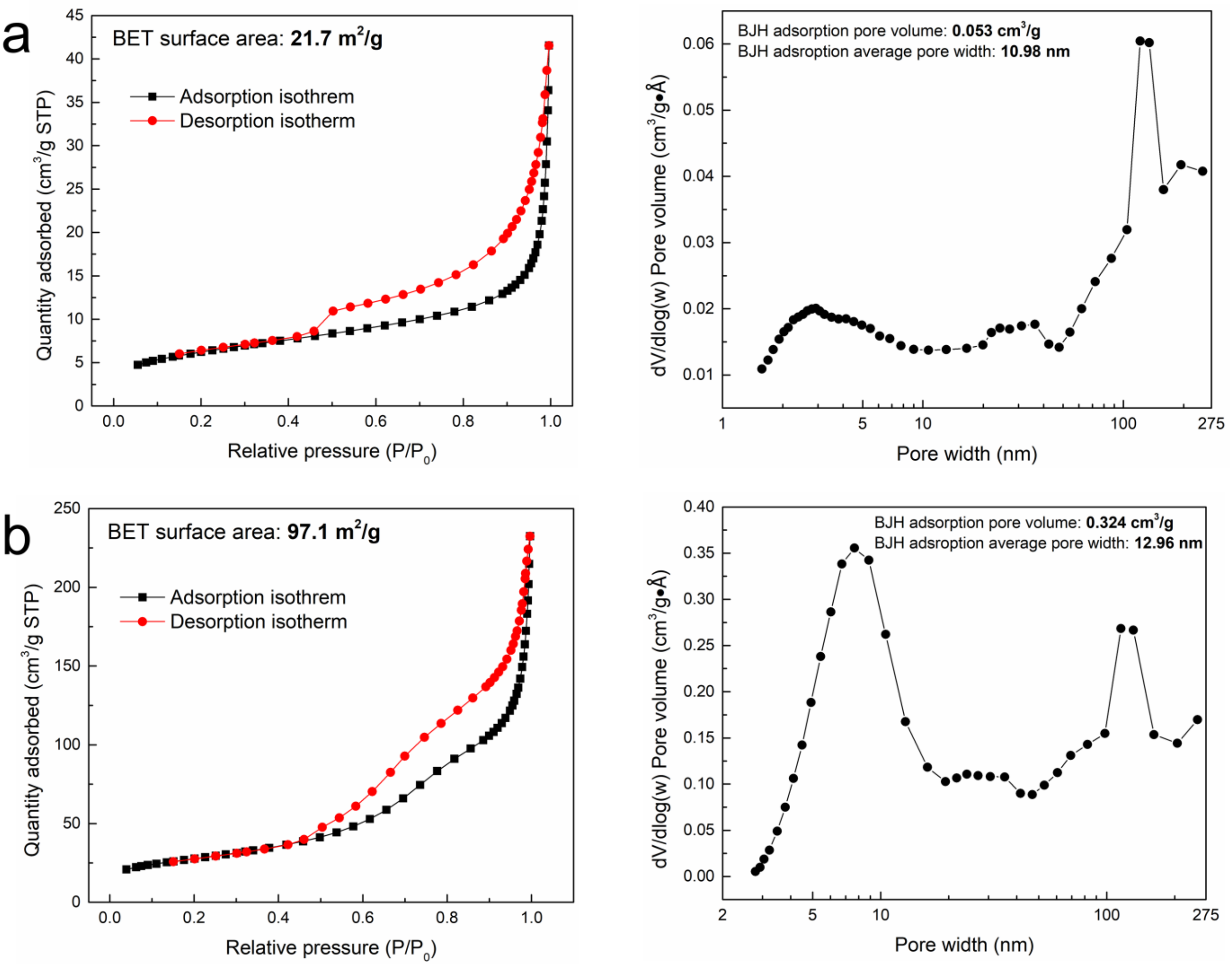

Supplementary Fig. 24. N_2 adsorption/desorption isotherms and BJH adsorption pore width distribution curves of flame synthesized **a.** non-porous $(Ni_{0.07}Al_{0.93})O_x$ solid solution and **b.** porous $(Ni_{0.07}Al_{0.93})O_x$ solid solution.

Compared to the non-porous $(Ni_{0.07}Al_{0.93})O_x$ solid solution nanoshell prepared by a the flame route without CTAB addition, the Brunauer–Emmett–Teller (BET) surface area increased from 21.7 to 97.1 m^2/g, and the pore volume increased from 0.053 to 0.324 cm^3/g. The Barrett–Joyner–Halenda (BJH) pore size analysis showed a mesopore size distribution of 3~20 nm. The small peak above 100 nm is likely due to the central voids in the hollow particles.

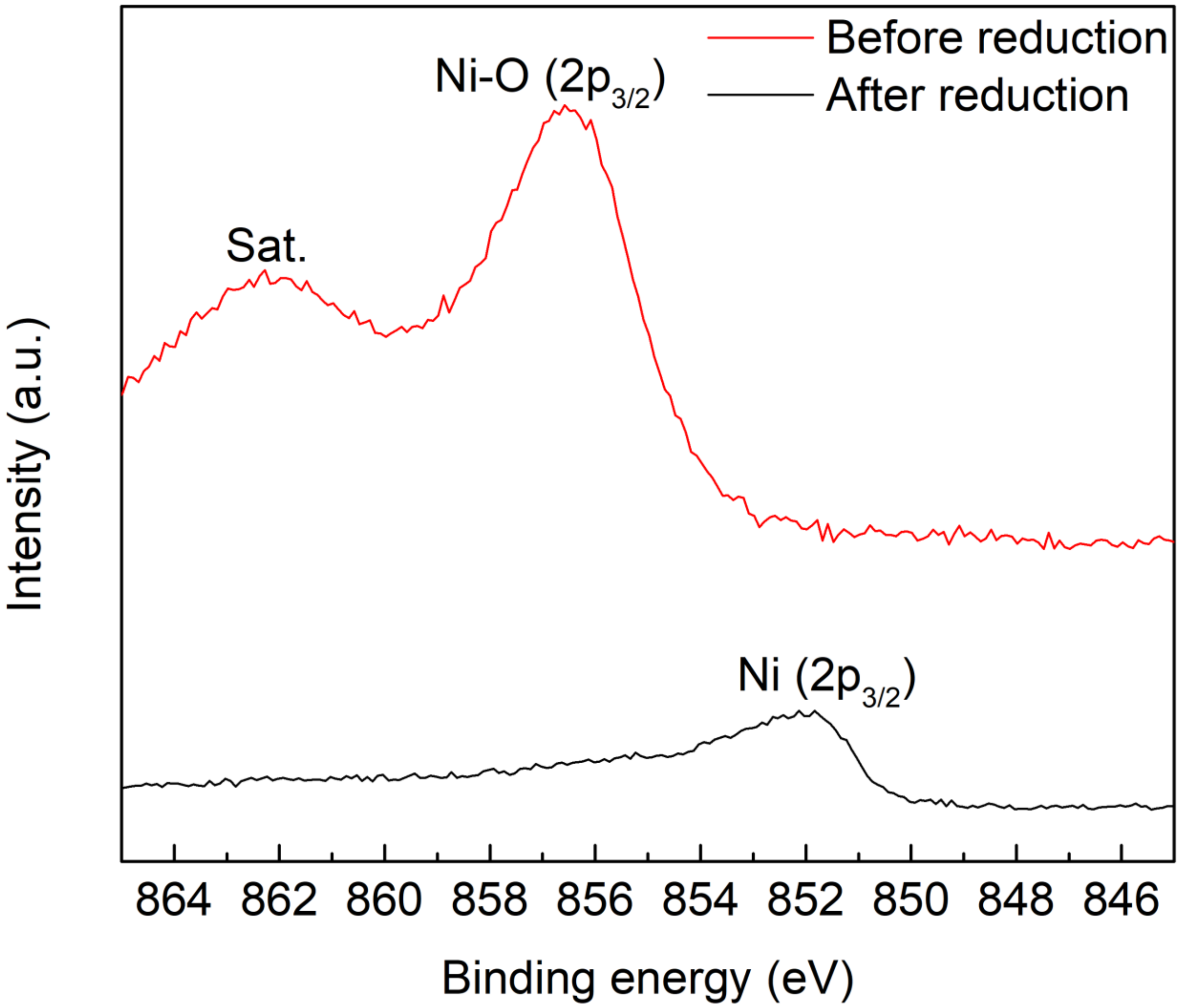

Supplementary Fig. 25. Ni *2p* XPS profiles of fresh flame synthesized $(Ni_{0.07}Al_{0.93})O_x$ and Ni/Al_2O_3 after H_2 reduction.

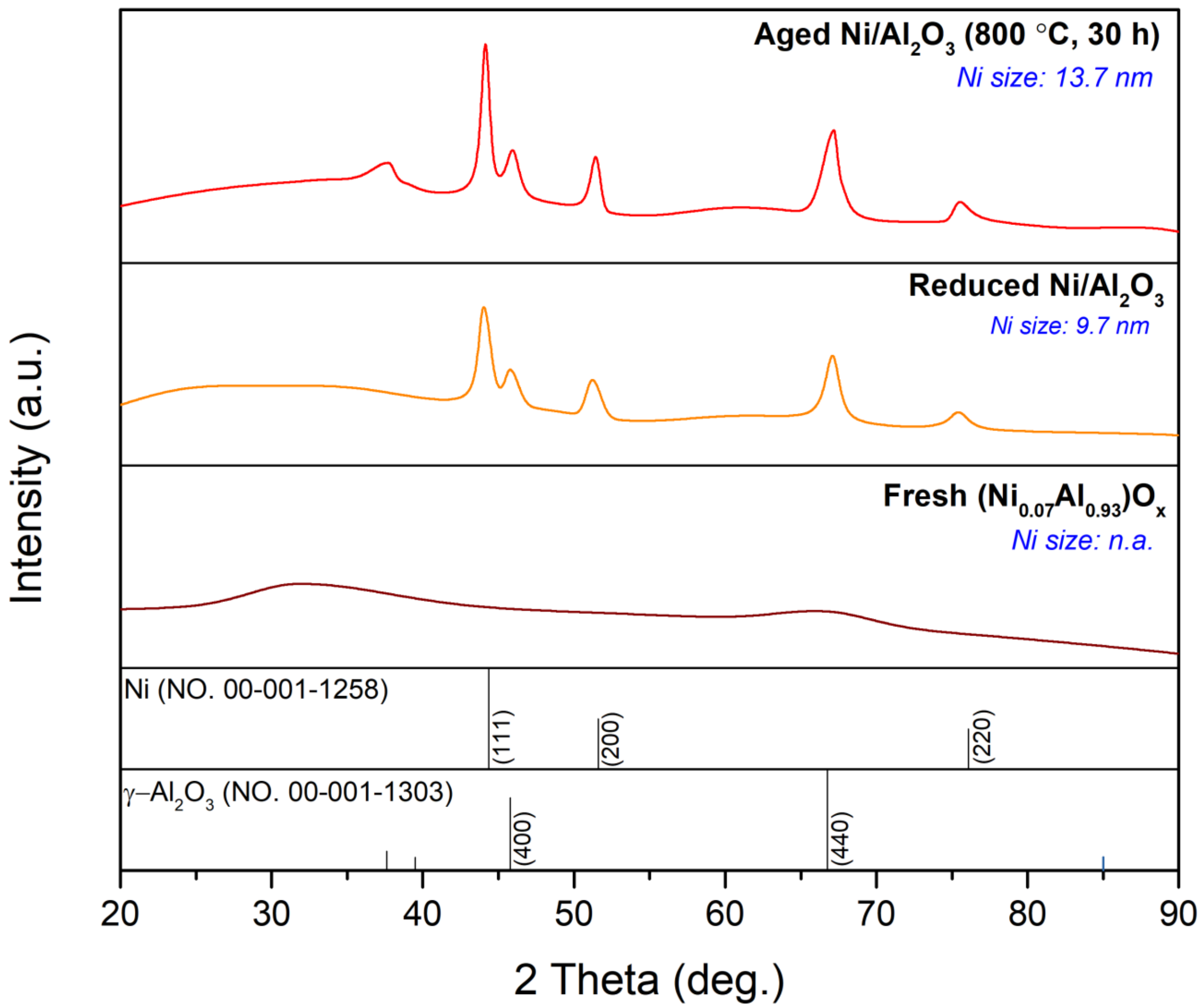

Supplementary Fig. 26. XRD patterns of fresh $(Ni_{0.07}Al_{0.93})O_x$, Ni/Al_2O_3 after H_2 reduction, and aged Ni/Al_2O_3 held at 800 ºC for 30 hours. The particle size was calculated using the Scherrer equation applied to the Ni (111) peak.

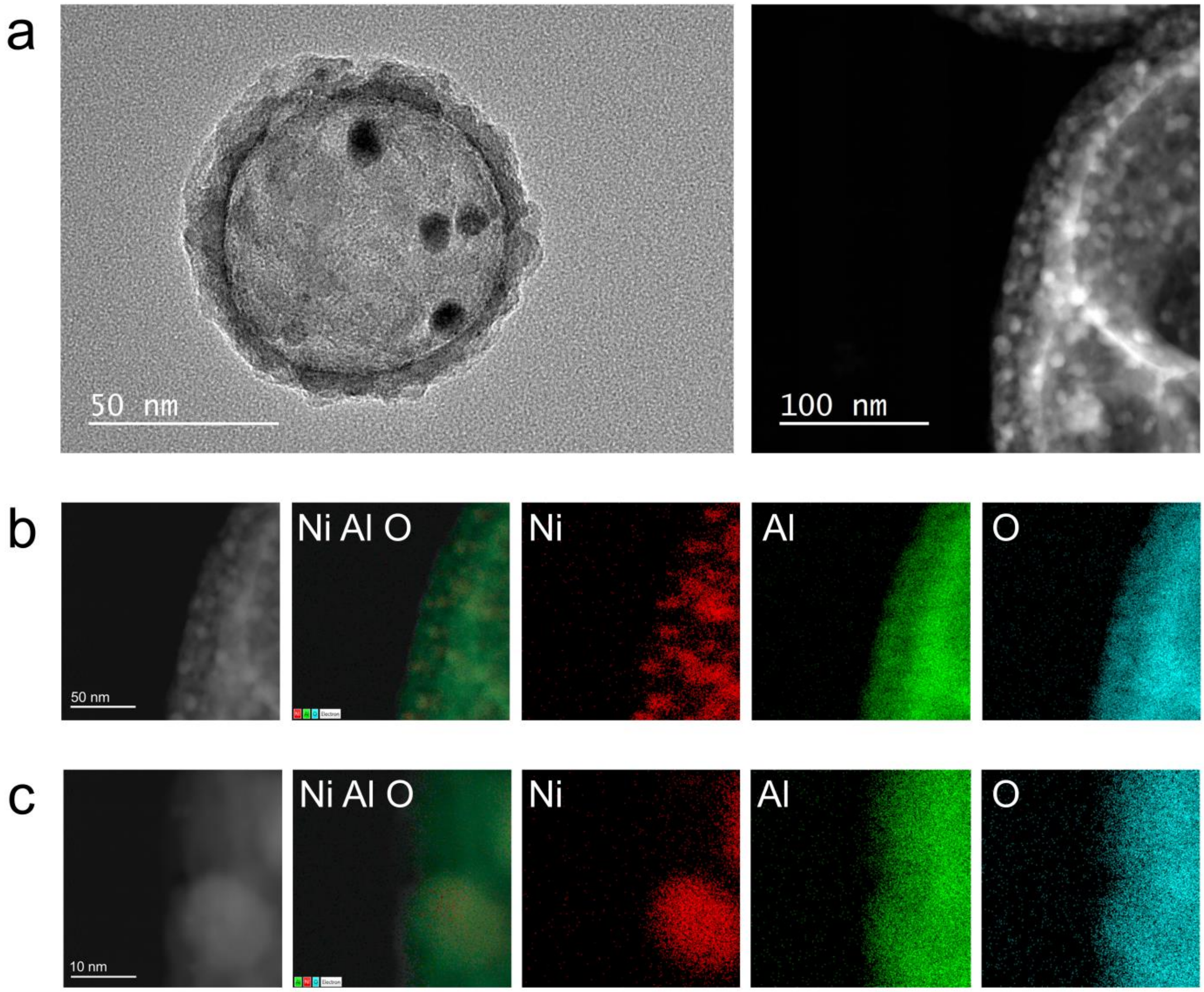

Supplementary Fig. 27. TEM, HAADF-STEM image and elemental maps of the Ni/Al_2O_3 after aging at 800 ºC for 30 hours.

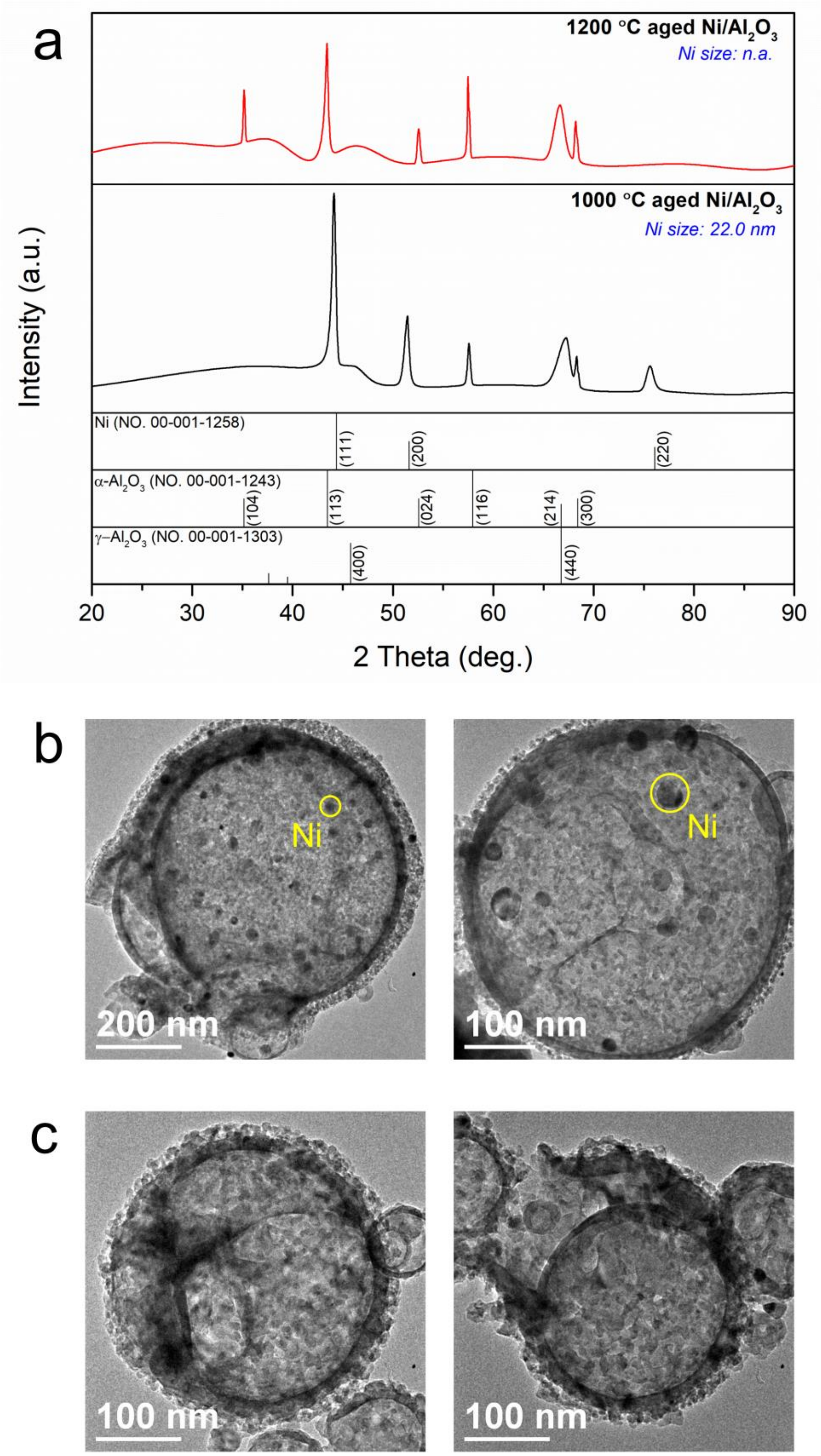

Supplementary Fig. 28. a. XRD patterns of the Ni/Al_2O_3 aged at 1000 ºC and 1200 ºC for 2 hours; TEM images of the Ni/Al_2O_3 aged at **b.** 1000 ºC and **c.** 1200 ºC for 2 hours.

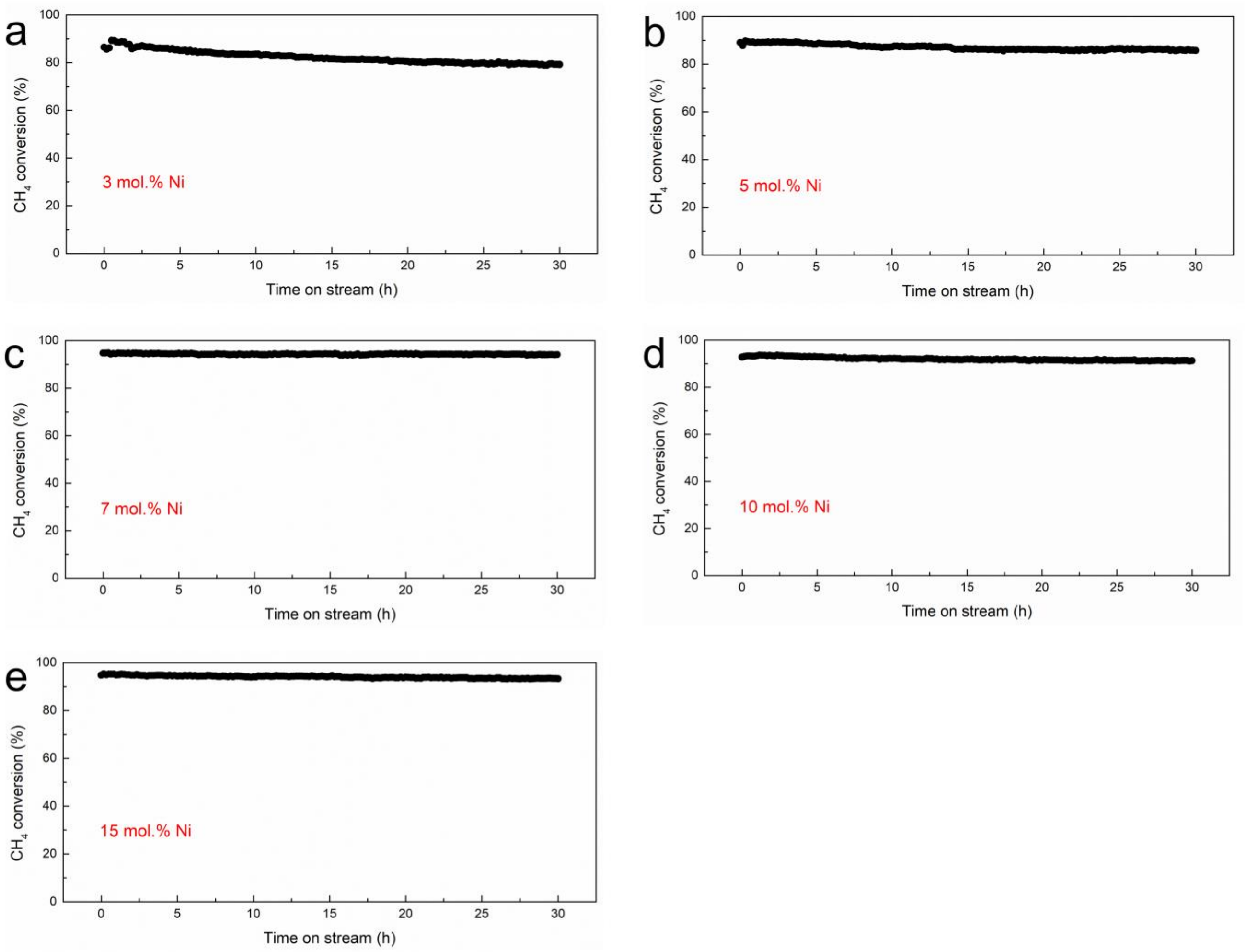

Supplementary Fig. 29. CH_4 conversions achieved using F-Ni/Al_2O_3 catalysts at 800 ºC with varied Ni content, corresponding to Fig. 6b.

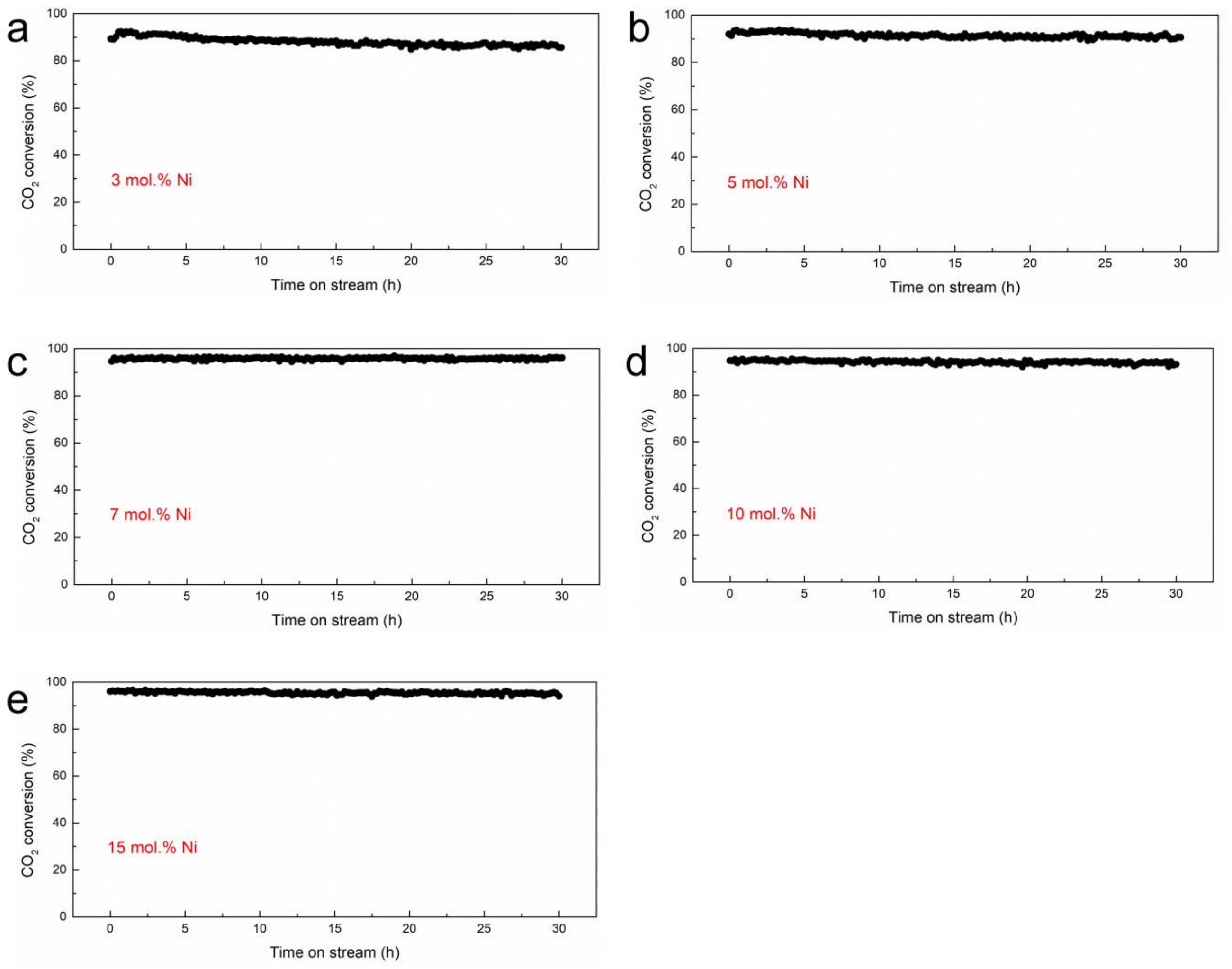

Supplementary Fig. 30. CO_2 conversions achieved using F-Ni/Al_2O_3 catalyst at 800 ºC with varied Ni content, corresponding to Fig. 6c.

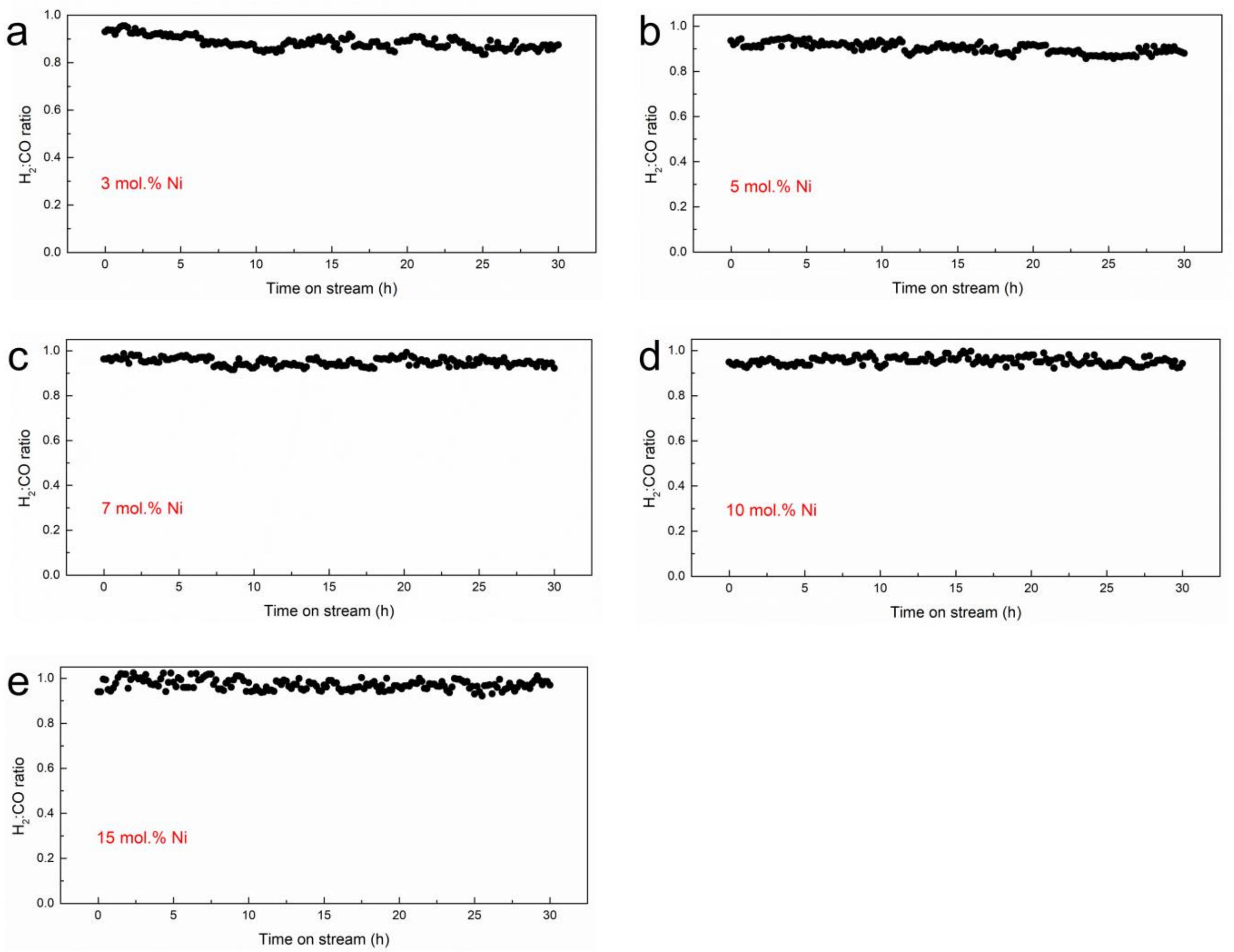

Supplementary Fig. 31. H_2:CO ratios achieved using F-Ni/Al_2O_3 catalyst at 800 ºC with varied Ni content, corresponding to Fig. 6d.

Supplementary Table 1. Rietveld refined XRD peak information of CP-NiO/ZrO_2 shown in Fig. 2G.

No.	2-theta (deg)	d (Å)	Height (cps)	FWHM (deg)	Size (Å)
1	30.47	2.931	885.7	0.41	209
2	35.35	2.537	176.9	0.41	213
3	37.25	2.412	19.9	0.80	110
4	43.48	2.079	39.6	1.24	72
5	50.73	1.798	396.6	0.50	184
6	60.29	1.534	230.1	0.59	162
7	63.30	1.468	49.2	0.92	106
8	74.56	1.272	34.0	0.92	114
9	82.52	1.168	50.1	0.93	119
10	85.06	1.140	33.2	0.73	154

Supplementary Table 2. Rietveld refined XRD peak information of F-$(Ni_{0.2}Zr_{0.8})O_x$ shown in Fig. 2G.

No.	2-theta (deg)	d (Å)	Height (cps)	FWHM (deg)	Size (Å)
1	30.60	2.919	136.6	0.82	105
2	35.44	2.531	31.1	0.69	127
3	50.99	1.790	61.7	1.03	90
4	60.62	1.526	40.6	1.05	91
5	63.55	1.463	6.5	1.53	64
6	82.81	1.165	6.7	1.46	76
7	85.48	1.135	4.7	2.49	45

Supplementary References

1. Araujo, T.P. et al. Flame-made ternary Pd-In_2O_3-ZrO_2 catalyst with enhanced oxygen vacancy generation for CO_2 hydrogenation to methanol. *Nat. Commun.* **13**, 5610 (2022).

2. Elrefaie, F. & Smeltzer, W. Thermodynamics of Nickel-Aluminum-Oxygen system between 900 and 1400 K. *J. Electrochem. Soc.* **128**, 2237 (1981).

3. Koirala, R., Pratsinis, S.E. & Baiker, A. Synthesis of catalytic materials in flames: opportunities and challenges. *Chem. Soc. Rev.* **45**, 3053-3068 (2016).

4. Yao, Y.G. et al. Carbothermal shock synthesis of high-entropy-alloy nanoparticles. *Science* **359**, 1489-1494 (2018).

5. Liu, X. et al. Strong Interaction over Ru/Defects-Rich Aluminium Oxide Boosts Photothermal CO_2 Methanation via Microchannel Flow-Type System. *Adv. Energy Mater.* **12**, 2201009 (2022).
